# The Administration of Circulating Extracellular Vesicles Modified by Anesthesia and Surgery Induces Delirium‐Like Behaviors in Aged Mice

**DOI:** 10.1111/cns.70483

**Published:** 2025-06-19

**Authors:** Yubo Gao, Shaling Tang, Jun Liu, Xiaoxia Yang, Hui Chen, Jiahui Li, Xinli Ni

**Affiliations:** ^1^ Department of Anesthesiology and Perioperative Medicine General Hospital of Ningxia Medical University Yinchuan China

**Keywords:** aged, anesthesia, circulating extracellular vesicles, miRNA, postoperative delirium, proteomic, surgery

## Abstract

**Background:**

The mechanisms by which peripheral surgery remotely affects brain function remain unclear. Circulating extracellular vesicles (EVs) are a complex membrane vesicle system composed of peripheral immune cells and other tissue sources. They can carry proteins and nucleic acids without being restricted by the blood–brain barrier. They transfer peripheral pro‐inflammatory factors and damage proteins to the brain, regulate intracellular signaling by acting on specific cells, and therefore play a role in cell‐to‐cell communication. It remains uncertain whether postoperative delirium occurs after anesthesia and surgery because peripheral blood EVs transfer peripheral pathogenic factors into the brain, inducing pathological changes in neuronal cells and cognitive and behavioral abnormalities. This study aimed to investigate the effects of circulating EVs from mice under anesthesia and surgery on the cognitive behavior and neuronal cells of recipient mice through animal experiments. Moreover, the expression profiles of differential proteins and microRNAs were analyzed in circulating EVs from mice undergoing anesthesia and surgery.

**Methods:**

We used aged mice and performed laparotomy under sevoflurane anesthesia to simulate the clinical environment. Twenty‐four hours after anesthesia and surgery, circulating EVs were extracted and injected into the control mice via the tail vein to observe cognitive behaviors and neuronal pathological changes in the recipient mice. To further identify the key pathogenic factors in circulating EVs, we used high‐throughput technology to detect the differential proteins and miRNAs in the circulating EVs of mice undergoing anesthesia and surgery compared to control mice. Candidate differential proteins and miRNAs were then screened and verified.

**Results:**

The administration of anesthesia and surgery‐derived EVs to control mice led to the manifestation of delirium‐like behavioral changes and pathological changes in nerve cells in recipient mice. SAA1, miR‐103‐3p, and miR‐31‐5p are potential target molecules implicated in POD.

**Conclusions:**

Circulating EVs are novel potential mediators involved in POD.

## Graphical abstract

The study indicates that the administration of anesthesia and surgery derived extracellular vesicles (EVs) to normal mice leads to the manifestation of delirium‐like behavioral changes, elevated microglial cell count, secretion of proinflammatory factors, and reduction in synaptic spine density in the recipient mice. SAA1, miR‐103‐3p, and miR‐31‐5p are potential target molecules implicated in POD. The findings suggest that circulating EVs are recognized as potential novel mediators that have a role in POD.

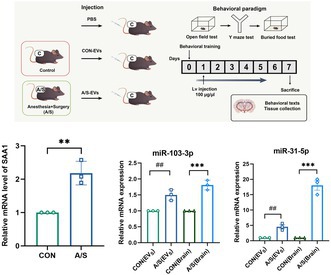

**Abbreviations**
A/S group: anesthesia and surgery group
BBB: blood–brain barrier
CNS: central nervous system
CON group: control group
DAPI: 40,6‐diamino‐2‐phenylene lindane
EVs: extracellular vesicles
FDR: false discovery rate
GO: Gene Ontology
KEGG: kyoto encyclopedia of genes and genomes
LPS: lipopolysaccharide
NTA: nanoparticle tracking analysis
PCA: principal component analysis
POCD: postoperative cognitive dysfunction
POD: postoperative delirium
PSD: postsynaptic density
RT‐PCR: real‐time fluorescence quantitative PCR
TEM: transmission electron microscopy

## 1 Introduction

Postoperative delirium (POD) is frequently encountered acute cognitive impairment observed in elderly individuals after administering anesthesia and surgical procedures. The condition is generally distinguished by a decline in memory function and changes in consciousness, typically manifesting within 24–72 h postoperatively [1]. Although its occurrence in the general population is approximately 2.5%–3%, it can exceed 50% in aged and high‐risk individuals. Moreover, the likelihood of experiencing this phenomenon seems to increase with age [2]. POD extends the duration of hospitalization and substantially escalates healthcare costs but also increases mortality risk within 30 days by at least 7%–10% [1]. With the ongoing global demographic shift towards an aging population, along with advancements in surgical techniques and anesthesia, there has been a notable rise in the relative and absolute numbers of elderly individuals undergoing surgical procedures. Consequently, this trend has resulted in a substantial increase in the number of POD cases [3]. However, POD pathogenesis remains unclear, and effective measures for prevention, diagnosis, and treatment are currently lacking [1]. Hence, it is imperative to conduct comprehensive research on the underlying causes of POD and to explore efficacious preventive and therapeutic approaches. These endeavors are of utmost importance in the context of the global aging population.

Numerous studies have indicated that the acute neuroinflammatory response triggered by surgical anesthesia and trauma may play a critical pathogenic role in POD [4]. The peripheral immune system can be quickly activated by surgical anesthesia and trauma, releasing several inflammatory and cellular components. Inflammatory factors can infiltrate brain tissue by multiple paths, including direct passage across the blood–brain barrier (BBB), disruption of the BBB, or transmission of peripheral immunological signals to the brain. Consequently, these processes alter the homeostasis of the central immune system [5]. Elevated pro‐inflammatory cytokine levels in the brain can lead to excessive microglial cell activation, producing additional pro‐inflammatory factors, complement, reactive oxygen species, and initiating inflammatory reactions within the central nervous system (CNS). This, in turn, results in neuronal damage, synaptic plasticity loss, and detrimental effects on cognitive function [6]. The presence of elevated pro‐inflammatory cytokine levels in the brain can lead to excessive microglial cell activation, resulting in the production of additional pro‐inflammatory factors, complement, reactive oxygen species, and the initiation of inflammatory reactions within the CNS [7]. These findings indicate a potential correlation between the peripheral immune system and the CNS. However, the specific routes through which peripheral inflammatory factors and toxic substances are transmitted to the brain and their interactions with neuronal cells leading to cognitive dysfunction remain unidentified.

Circulating extracellular vesicles (EVs) are complex membrane vesicles composed of peripheral immune cells and other tissue sources [8]. These vesicles transport biologically active molecules, including proteins, microRNAs (miRNAs), nucleic acids, and lipids. Through blood circulation, they can reach distant target tissues and organs, including the brain, where they exert their effects on target cells. EVs are vital in intercellular communication by rapidly regulating intracellular signals [9, 10]. Previous studies suggest that circulating EVs are important peripheral‐to‐brain communication mediators [11] and can transmit pathological effects from the periphery to the brain [12, 13]. Some studies identified that surgical trauma and anesthetic drugs can alter the cargo of circulating EVs [14, 15]. However, the role of circulating EVs in POD development has not yet been evaluated.

In this study, by focusing on circulating EVs, we observed the effects of circulating EVs from mice with anesthesia and surgery on the cognitive behavior and neuronal cells of recipient mice through animal experiments. The expression profiles of differential proteins and miRNAs in circulating EVs from mice undergoing anesthesia and surgery, as well as control mice, were analyzed using high‐throughput sequencing. Candidate proteins and miRNAs were screened and verified, offering theoretical and experimental evidence for investigating the POD mechanism and identifying potential new biomarkers and targets.

## 2 Materials and Methods

### 2.1 Animals

Wild‐type male C57BL/6J mice (16–18 months of age) were used for all experiments. Mice were purchased from Beijing Viton Lihua Laboratory Animal Technology Company (Beijing, China) at 10 months of age. All mice were housed in a 12‐h light/dark cycle and fed ad libitum standard rodent chow and water.

All experiments were approved by the Medical Research Ethics Review Committee of the General Hospital of Ningxia Medical University Hospital (ethics no. KYLL‐2023‐0183).

### 2.2 Anesthesia and Surgery

The mice in the anesthesia and surgery group (A/S group) underwent laparotomy under anesthesia with sevoflurane. Anesthesia induction was performed using 5% sevoflurane in a closed anesthesia induction chamber for 3 min. After anesthesia induction, the mice were placed in a supine position and secured on the operating table, and surgery was performed under 2% sevoflurane. A midline abdominal incision of approximately 3 cm was made to expose the surgical field. Sterile cotton swabs soaked in normal saline were used to gently explore abdominal organs, including the liver, spleen, kidneys, stomach, large intestine, and small intestine. The intestines were gently manipulated on a sterile gauze pad moistened with physiological saline. The entire procedure lasted for 30 min. Muscles, fascia, and skin were sutured with 5‐0 nylon thread. Postoperatively, 0.2 mL of 0.2% ropivacaine was injected subcutaneously along the incision for analgesia. Sevoflurane anesthesia was terminated postoperatively, and the mice were returned to their cages. During the surgical procedure, a heating pad was used to maintain a temperature of 37°C. The control group (CON group) of mice did not undergo any anesthesia or surgery.

### 2.3 EVs Isolation, Quantification, Morphology, Marker Proteins Analysis and Labeling

Mice in the A/S group and CON group were deeply anesthetized with sevoflurane 24 h after surgery, and blood was collected from the left ventricle using a 23‐gauge needle attached to a 2 mL syringe, then immediately transferred into 1.5 mL Eppendorf tubes. Blood was allowed to clot at room temperature for 45 min, followed by centrifugation at 3000 *g* for 10 min at 4°C. The supernatant (serum) was stored at –80°C until further use.

#### 2.3.1 Circulating EVs Isolation

EVs were isolated using the ExoQuick kit (EXOQ5A‐1; SBI, USA) according to the manufacturer's instructions. Collected mouse serum was centrifuged to remove cells and cell debris. The supernatant was collected and filtered through a 0.22 μm sterile filter into a sterile container. ExoQuick Exosome Precipitation Solution was added to the supernatant at a 4:1 ratio, thoroughly mixed and then incubated at 4°C refrigerator for 30 min. After centrifugation, the supernatant was aspirated, leaving a beige or white pellet at the bottom of the tube. The pellet was resuspended in 100 μL of PBS and stored at –80°C.

#### 2.3.2 Nanoparticle Tracking Analysis (NTA)

The size and particle concentration of the circulating EVs were determined by NTA using a NanoSight NS300 (Sysmex Belgium). Samples were diluted in sterile‐filtered PBS to achieve a range of 20–50 particles per frame. The sample was injected into the sample chamber and manually measured five times at 25°C, with five consecutive videos being recorded. The averages of the five consecutive videos for each sample were calculated and later used for result visualization in terms of concentration (particles/mL) and particle size (nm).

#### 2.3.3 Transmission Electron Microscopy (TEM)

A total of 3 μL of freshly isolated EVs were loaded onto copper grids and left in place for 1–2 min. The excess vesicle suspension was carefully removed using filter paper. Then, use a disposable Pasteur pipette to apply a small amount of 1% phosphotungstic acid solution onto the prepared sample on the copper grid. After 30 s, the excess negative stain solution was gently removed from the side using filter paper. The copper grids were washed three to five times with distilled water, and after careful removal of excess water using filter paper, they were allowed to air dry at room temperature. The EVs morphology was observed using a TEM (Hitachi H‐7700).

#### 2.3.4 Immunoblot Analysis of EVs Marker Proteins

Total protein was extracted from serum‐derived EVs using RIPA buffer, and quantification was performed using a BCA assay kit (Beyotime, China). Twenty microgram of total protein extracted from EVs were subjected to electrophoresis. Antibodies against the EVs markers, CD63 (Abcam, 1:600), CD9 (Abcam, 1:600), and Calnexin (Cell Signaling Technology, 1:500), were used for overnight blotting. Detection was carried out using anti‐rabbit IgG secondary antibody (Cell Signaling Technology, 1:10,000), and immunoblotting results were observed using chemiluminescence and a ChemiDoc system (Bio‐Rad, UK).

#### 2.3.5 Labeling of EVs

EVs were labeled with PKH26 (MKCN8501; Sigma, USA) by adding 1 mL of PKH26 dye solution C to the EVs and mixing well, after incubation for 5 min, an equal volume of 10% fetal bovine serum was added to terminate the labeling. The mixture was centrifuged at 100,000 × *g* for 70 min, the supernatant was discarded, and the pellet was resuspended in sterile 1× PBS. PKH26‐labeled EVs (100 μL)were injected into normal aged mice via the tail vein, and brain tissues were collected 24 h later for fluorescence staining to observe the distribution and expression of EVs in the brain.

### 2.4 Tail Vein Injection of Circulating EVs and Experimental Grouping

Tail vein injections were performed using a mouse tail vein injection viewer (GD‐MI, Nanjing, China). Male C57BL6 mice (16–18 months old, 20 mice per group) were subjected to tail vein injections: CON‐EV_S_ group received EVs isolated from normal aged mice, A/S‐EV_S_ group received EVs isolated from mice after anesthesia and surgery (100 μg/μL), and PBS group received an injection of an equal volume of PBS (100 μL) as the control group. Injections were administered daily for 5 days and behavioral tests were performed on the 7th day.

### 2.5 Behavioral Tests

All behavioral experiments were conducted in the morning and recorded by a researcher who was unaware of the grouping of the animals. The instruments were cleaned with 75% alcohol before each test to avoid olfactory interference.

#### 2.5.1 Buried Food Test

The test mice received two pieces of sweet cereal 1 day before the test. The cages with clean bedding were placed in a quiet room 1 h before the test to acclimate to the test environment. The grains were buried 0.5 cm below the bed to prevent the rats from seeing the grains directly, and then the rats were placed in the middle of the cage. The latency to feeding (time from cage entry to discovery and grain consumption) was recorded. The observation time was 5 min. If the mice did not find grain in 5 min, the latency was recorded at 300 s.

#### 2.5.2 Open Field Test

Each mouse was placed in an open field chamber (40 cm × 40 cm × 40 cm) under dim lighting and allowed to move freely. The trajectory was recorded for 5 min, and the following parameters were recorded using a video monitoring analysis system (Panlab SMART 3.0): total distance, center distance, entry into the center, time spent in the center, and total freezing time.

#### 2.5.3 Y‐Maze Experiment

The Y‐maze was composed of three identical arms (8 cm × 30 cm × 15 cm) with an angle of 120° between each arm. The three arms were randomly defined as: the start arm, that is, the arm in which the mice started to enter the maze, which was always open during the experiment; the other arms, which were always open during the experiment; and the new isolated arm, that is, the arm that was isolated during the training phase and opened during the testing phase. The experiment was divided into two phases (i.e., training and testing phases). During the training phase, mice were free to explore areas other than the newly isolated arm, starting from the starting arm; after 10 min, training was completed and the mice were returned to the rearing cage. After 2 h, the mice were placed in the testing phase and freely explored the three groups for 5 min. Mouse activity was tracked using animal behavior analysis software to record the number of arm visits, the duration of new arm visits, and the number of new arm visits.

### 2.6 Immunofluorescence Staining

Mice were deeply anesthetized with sevoflurane and perfused with 0.1 M PBS. The brains were removed, fixed in 4% paraformaldehyde overnight at 4°C, and subsequently soaked in 20% and 30% sucrose before cryoprotecting. The frozen brains were cut into 20 μm coronal sections using a cryosectioner and stored in cryoprotectant (30% ethylene glycol, 30% sucrose, and 0.02 M PB) at −20°C until use. Brain sections were washed with PBS, treated with 3% hydrogen peroxide, and blocked and permeabilized with 10% goat serum and 0.3% Triton X‐100. The sections were then incubated overnight with primary antibodies Iba‐1 (Wako 1:500, 019‐1974 or Abcam 1:500, ab5076 and GFAP 1:500, Abcam, respectively) and CD86 (Biolegend 1:500, 105007) incubated with secondary antibody (goat anti‐rabbit, 1:2000, ThermoFisher) with fluorescent dye 488 for 2 h at room temperature in the dark. Immunolabeled sections were covered with 40,6‐diamino‐2‐phenylene lindane (DAPI). Histological images were observed with a fluorescence microscope.

Each group randomly selected 5 mice, and three brain slices were selected from each mouse. The number of Iba‐1 positive cells in the hippocampal CA1 area was imaged using a fluorescence microscope, representing microglia numbers. As previously described [16], Image J was used to analyze the morphological indicators of microglia. Fifteen fields of view were selected for each group, and three microglia were selected from each field of view. Only cells with the cell body and projections entirely included in the slice were included in the analysis. Images were analyzed using Image J according to the following steps: setting the threshold to visualize all processes, removing noise, converting the image to a binary image, using the skeletonization plugin to skeletonize microglia, and observing the branch density of microglia. The soma area and arborization area of each microglia were measured using Image J. The soma area was determined by drawing a line around the cell body using the freehand selection tool, drawing a region at the distal end of each microglia branch using the polygonal selection tool, measuring the area of that region as the arborization area, and calculating the morphological index of microglia (soma area/arborization area) to more accurately assess the morphological characteristics of the cells and thus quantify the degree of microglia activation.

### 2.7 Hippocampal Tissue Western Blotting

Hippocampal tissues were isolated, and total proteins were extracted using a total protein extraction kit (Beyotime Biotech Inc., China). The protein concentration of each sample was measured using the BCA (Beyotime Biotech Inc., China). The extracted protein samples (20 μg) were added to the wells of a 10% SDS‐PAGE gel for electrophoresis separation, and then the separated proteins in the gel were transferred to a polyvinylidene fluoride membrane. The membrane was incubated in diluted primary antibody solution overnight. Primary antibody dilution concentration: Iba‐1 (1:1000; Abcam). Then, the membrane was placed in the corresponding secondary antibody dilution and incubated on a shaker at room temperature for 2 h, followed by membrane washing. Luminous liquid was applied to the incubated membrane, exposed and photographed, and analyzed with Image J.

### 2.8 Real‐Time Fluorescence Quantitative PCR (RT‐PCR)

Tissues from the hippocampal region of the brain were collected according to the experimental design, and total RNA was extracted using TRIzol reagent (DP419; Tiangen, China). Total RNA was reversed to cDNA using the BTransScript First Strand cDNA Synthesis Kit (AU341‐02; Beijing All Style Gold Biotechnology Co. Ltd., China). PCR was performed using 2× SYBR green qPCR premix (aq601‐03; Beijing All Style Gold Biotech Co. Ltd., China). GAPDH was used as an internal reference. The relative mRNA expression of genes was statistically analyzed using the $2^{-∆∆C_{t}}$ method. All primer sequences are listed in Table 1.

**TABLE 1:** Primer sequences used for quantitative RT‐PCR SYBR Green protocol.

Primer	Forward	Reverse
IL‐1β	CACTACAGGCTCCGAGATGAACAAC	TGTCGTTGCTTGGTTCTCCTTGTAC
IL‐6	CTTCTTGGGACTGATGCTGGTGAC	TCTGTTGGGAGTGGTATCCTCTGTG
TNF‐α	CACCACGCTCTTCTGTCTACTGAAC	AGATGATCTGAGTGTGAGGGTCTGG
CD86	GACACCCACGGGATCAATTA	GCCTCCTCTATTTCAGGTTCAC
SAA1	GACACCATTGCTGACCAGGAA	GGCAGTCCAGGAGGTCTGTAG
miR‐103‐3p	GCAGCAUUGUACAGGGCUAUGA	UCAUAGCCCUGUACAAUGCUGCU
miR‐31‐5p	AGGCAAGAUGCUGGCAUAGCUG	CAGCUAUGCCAGCAUCUUGCCU
miR‐107‐3p	AGCAGCAUUGUACAGGGCUAUCA	UGAUAGCCCUGUACAAUGCUGCU
GAPDH	AGGAGCGAGACCCCACTAACA	AGGGGGGCTAAGCAGTTGGT

### 2.9 ELISA

Hippocampal tissue was extracted, homogenized, and the supernatant was collected for analysis. ELISA kits were utilized to measure the expression levels of inflammatory factors IL‐1β, IL‐6, and TNF‐α in the hippocampal tissue (Interleukin 1 Beta ELISA Kit, ELK1271, ELK Biotechnology, Interleukin 6 ELISA Kit, ELK1157, ELK Biotechnology, Tumor Necrosis Factor Alpha ELISA Kit, ELK1387, ELK Biotechnology, China).

### 2.10 Golgi Staining and Analysis

Golgi staining was performed using the FD Rapid GolgiStain Kit (FD Neuro Technologies) following the provided instructions. In summary, the right half of the mouse brain was collected, rinsed with physiological saline, and then immersed in a 1:1 volume ratio of Solution A:Solution B impregnation at room temperature in the dark for 14 days (with a change of solution after 24 h). The brain was stored in Solution C at 4°C for 2 days (with one solution change after 12 h). The brain samples were then mounted on a vibratome stage and sliced at 100 μm. The samples were transferred to glass slides with a gelatin‐coated coverslip containing Solution C and left to dry for 3 days at room temperature. The staining procedure was as follows: the slides were rinsed twice with distilled water for 4 min each time, a mixed solution was prepared in a 1:1:2 ratio of Solution D, Solution E, and water, and the slides were immersed in this mixed solution for 10 min. Subsequently, we dehydrated it with a series of ethanol gradients, cleared it with xylene, and sealed it with mounting medium.

The dendritic complexity of pyramidal neurons and the density of spines on secondary dendrites in the CA1 area were analyzed using ImageJ. The branching was calculated using the Sholl 14 analysis. Concentric circles spaced 10 μm apart and centered on the torso were drawn, and the number of intersections with the concentric circles was quantified. The target area of the brain tissue was selected for 1000× imaging using an Eclipse Ci‐L photomicrographic microscope. After imaging was completed, the number of dendritic spines in the 30–90 μm length range of the 2nd or 3rd dendritic branch in the intact neuron in the center of each 1000× image was measured separately using Image‐Pro Plus 6.0 analysis software to measure the length and count the number of dendritic spines within that length, using the number of dendritic spines per 10 μm as its density = number of dendritic spines/dendritic length × 10.

### 2.11 Transmission Electron Microscope

The heart was perfused with physiological saline and 4% paraformaldehyde at 4°C, resulting in stiffness in the mouse's neck, limbs, and tail. Subsequently, the skull was swiftly removed using ophthalmic scissors and tweezers, and the hippocampal tissue was carefully extracted and identified under a stereomicroscope. The hippocampal CA1 region was then isolated and cut into 1 mm^3^ tissue blocks, which were placed in EP tubes containing 3% glutaraldehyde and stored at 4°C for 24 h. The tissue blocks were then washed three times with PBS for 10 min each. Following this, the hippocampal CA1 tissue underwent fixation, dehydration, and embedding. The embedded CA1 tissue was sliced into ultra‐thin sections (100 nm thick) and mounted on copper grids. Subsequently, the slices were double‐stained with uranyl acetate and lead citrate, and examined and photographed using an HT7700 transmission electron microscope. Each group consisted of 3 mice, with 15 randomly selected fields of view for synapse counting in each group. A total of 60 synapses were randomly chosen per group. The synaptic ultrastructure was quantified, and synapses were measured using Image J software. Analysis included assessing the average thickness of postsynaptic density (PSD) and the length of the synaptic active zone, as detailed in Ref. [17].

### 2.12 Proteomic Analysis of Circulating EVs

Circulating EVs were isolated from the blood of CON and A/S group mice, with six samples per group. The total proteins of the EVs were extracted by Shanghai Jikai Gene Medical Technology Co. Ltd. for proteomics analysis. The protein concentration of each EV was determined using the BCA method. For each sample, 20 μg of protein were mixed with six times the sample loading buffer and boiled for 5 min in a water bath. The samples were then subjected to 12% SDS‐PAGE electrophoresis at a constant voltage of 250 V for 40 min, followed by Coomassie Brilliant Blue staining. Peptide desalting was performed using a C18 cartridge and, after freeze drying, the peptides were reconstituted in 40 μL of 0.1% formic acid solution, followed by peptide quantification (OD280). Each sample was separated using a nanoflow Easy nLC system. After chromatographic separation, mass spectrometry analysis was performed using an Orbitrap Exploris 480 mass spectrometer. The raw mass spectrometry data were analyzed using MaxQuant software version 1.6.17.0. The mass spectrometry data were searched against a database with a global false discovery rate (FDR) cutoff value set at 0.01 for peptide and protein identification. The protein abundance was analyzed based on the normalized spectral protein intensity (LFQ intensity). Proteins with a fold change > 2 or < 0.5 and *p* value (*t*‐test) < 0.05 were considered differentially expressed proteins. Subcellular localization analysis was performed using WoLF PSORT (download link: https://wolfpsort.hgc.jp/). Domain prediction was carried out using InterProScan software. Gene Ontology (GO) annotation was performed using the GO database, and all differentially expressed proteins were functionally annotated using the Blast2GO software (www.geneontology.org). Pathway analysis was performed using the kyoto encyclopedia of genes and genomes (KEGG) database (https://www.kegg.jp/). Functional enrichment analysis was performed using Fisher's exact test.

### 2.13 Sequencing and Differential Expression Analysis of Circulating EVs miRNAs

Total RNA was extracted from the A/S group and the CON group using the exoRNeasy Serum/Plasma Maxi Kit (QIAGEN, Germany), with three samples per group. A 1 μL aliquot of RNA was taken for staining and the RNA concentration was measured on the Quantus Fluorometer. Based on the RNA concentration, the RNA was appropriately diluted with the matching diluent using NR1 cards and then subjected to machine detection. The analysis of circulating EVs miRNA sequencing was outsourced to Shanghai Jikai Gene Medical Technology Co. Ltd. The construction of a small RNA library was performed using the Illumina platform with the QIAseq miRNA Library Kit (Qiagen) kit. Differential expression analysis of miRNAs was performed using DESeq2. Differentially expressed miRNAs were identified as threshold *p* value < 0.05. The prediction of the target gene of differentially expressed miRNAs was performed using miranda, miRWalk, miRTarBase, and Targetscan. GO functional annotation analysis, Reactome, and KEGG pathway enrichment analysis were performed using the DAVID website to screen for enrichment analysis entries and pathway information.

### 2.14 Cell Culture and Transfection

The BV2 cell cryovial (Shanghai, China) was cultured with high‐glucose DMEM (Gibco) supplemented with 10% fetal bovine serum (FBS) and 1% penicillin–streptomycin. Place the flask in a 37°C incubator with 5% CO. Refresh the medium every 2–3 days. When cells reach 80%–90% confluency, perform routine subculture. Proceed with transfection when cells reach 60%–70% confluency. Reagent preparation: centrifuge FAM‐siRNA, mimics‐NC, and mimics‐miR‐103‐3p (General Biosystems Co. Ltd.) at 3000 rpm for 1 min. Resuspend and dilute the reagents to a final mimics concentration of 50 nM. The transfection was performed according to the manufacturer's instructions. After 4–6 h transfection, replace the medium with fresh complete medium containing serum. CON Group: BV2 cells cultured in fresh complete medium for 24 h. Mimics‐NC Group: BV2 cells transfected with mimics‐NC for 48 h. Mimics‐miR‐103‐3p Group: BV2 cells transfected with mimics‐miR‐103‐3p for 48 h. LPS Group: BV2 cells treated with 200 μg/mL lipopolysaccharide (LPS) for 24 h (positive control). Validation: Assess FAM‐siRNA fluorescence 48–72 h post‐transfection to confirm transfection efficiency.

### 2.15 CCK‐8 Assay

After treating cells under the experimental grouping conditions, seed cells from each group into a 96‐well plate. Following treatment, discard the supernatant and wash the cells once with PBS. Add 100 μL of pre‐prepared CCK‐8 working solution to each well. The working solution is prepared by mixing culture medium with CCK‐8 reagent (TransGen Biotech, Beijing) at a 10:1 (v/v) ratio. Incubate the plate in a cell culture incubator for 2 h. After incubation, measure the absorbance (OD) of each well at 450 nm using a microplate reader (Bio‐Rad). Calculate the cell viability for each group.

### 2.16 Statistical Analysis

Data analysis and plotting were performed using R software, SPSS (version 25.0), and GraphPad Prism (version 9.0). Data are presented as mean ± standard deviation. Normality was assessed using the Kolmogorov–Smirnov (K–S) test. Differences between the two groups were analyzed using the *t*‐test, whereas comparisons of categorical variables were conducted using the chi‐square or Fisher's exact tests. A one‐way analysis of variance was used for comparisons among multiple groups, followed by Tukey's post hoc test to examine differences between groups. In the statistical analysis, a *p* value < 0.05 was considered statistically significant.

## 3 Results

### 3.1 Characterization and Identification of Circulating EVs

**FIGURE 1:**
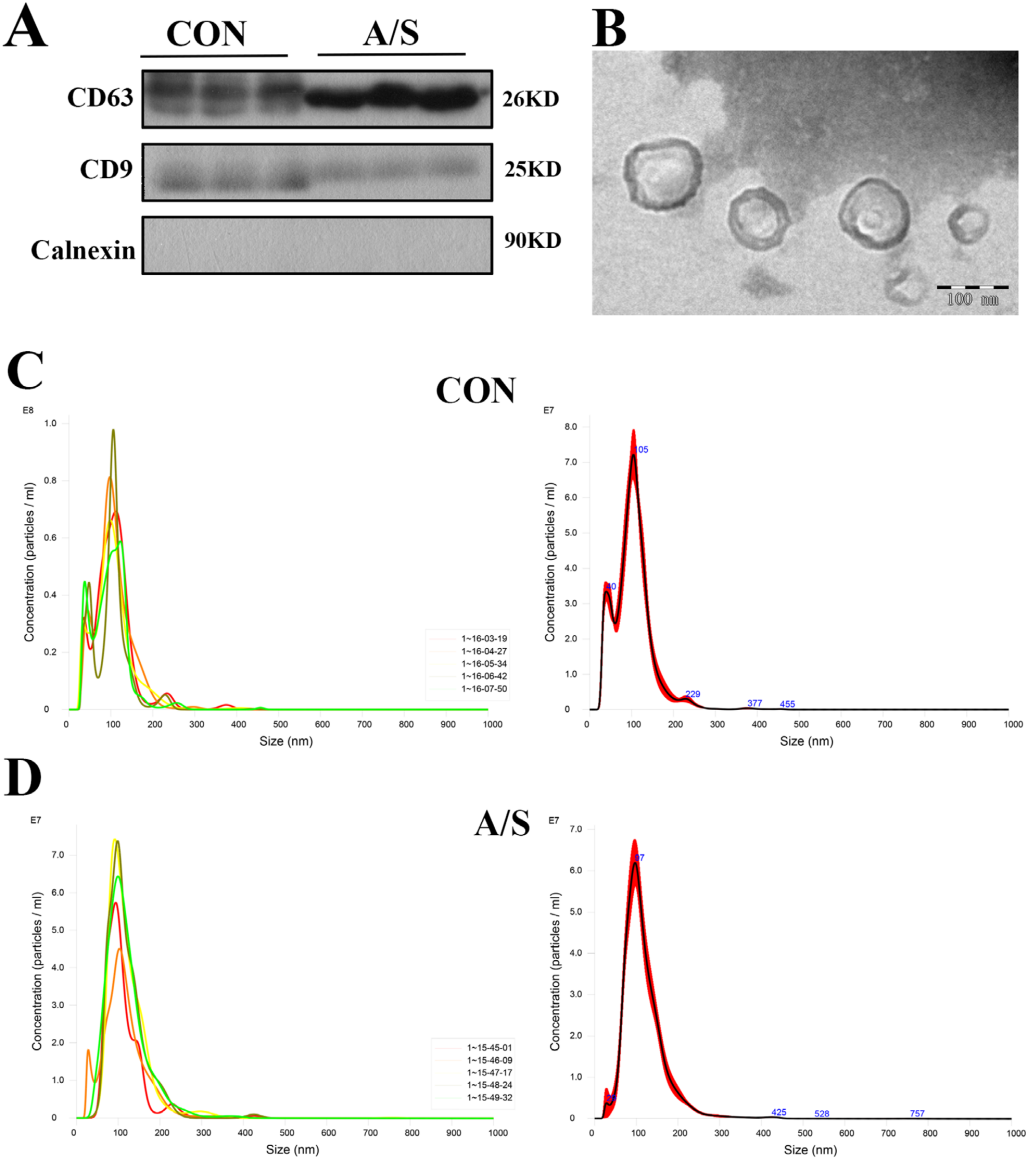
Characterization and identification of circulating EVs. (A) Western blotting detection of positive markers CD9 and CD63, and negative specific marker calnexin protein expression in circulating EVs in the A/S and CON groups of aged mice. (B) Representative TEM image depicts the typical morphology and size range of circulating EVs in aged mice after anesthesia and surgery. (C, D) Nanoparticle tracking analysis of the number and size distribution of circulating EVs in A/S and CON groups, the figure reveals the number and size of circulating EV_S_ for CON group (C) and A/S group (D), mean ± standard deviation (*n* = 5).

EVs were isolated from the serum of aged mice in the A/S and CON groups. A clear round membranous vesicle, typical of EVs, with a diameter of approximately 100 nm, was verified by transmission electron microscopy (TEM). We assessed the presence of typical EVs surface markers by western blotting and found that EVs in the A/S and CON groups were positive for CD63 and CD9 and negative for calnexin (Figure 1A). However, nonsignificant differences in morphology and size were observed between the A/S and CON groups under TEM (Figure 1B). Further analysis of the circulating EVs using nanoparticle tracking analysis (NTA) (Figure 1C,D) revealed a slightly larger average particle size in the A/S group (116.8 ± 1.9 nm) compared to the CON group (103.0 ± 1.6 nm). However, these differences in size were not large enough to be considered statistically significant, and both groups exhibited similar EVs concentrations.

### 3.2 Circulating EVs Crossing the BBB and Underwent Uptake by Microglia

**FIGURE 2:**
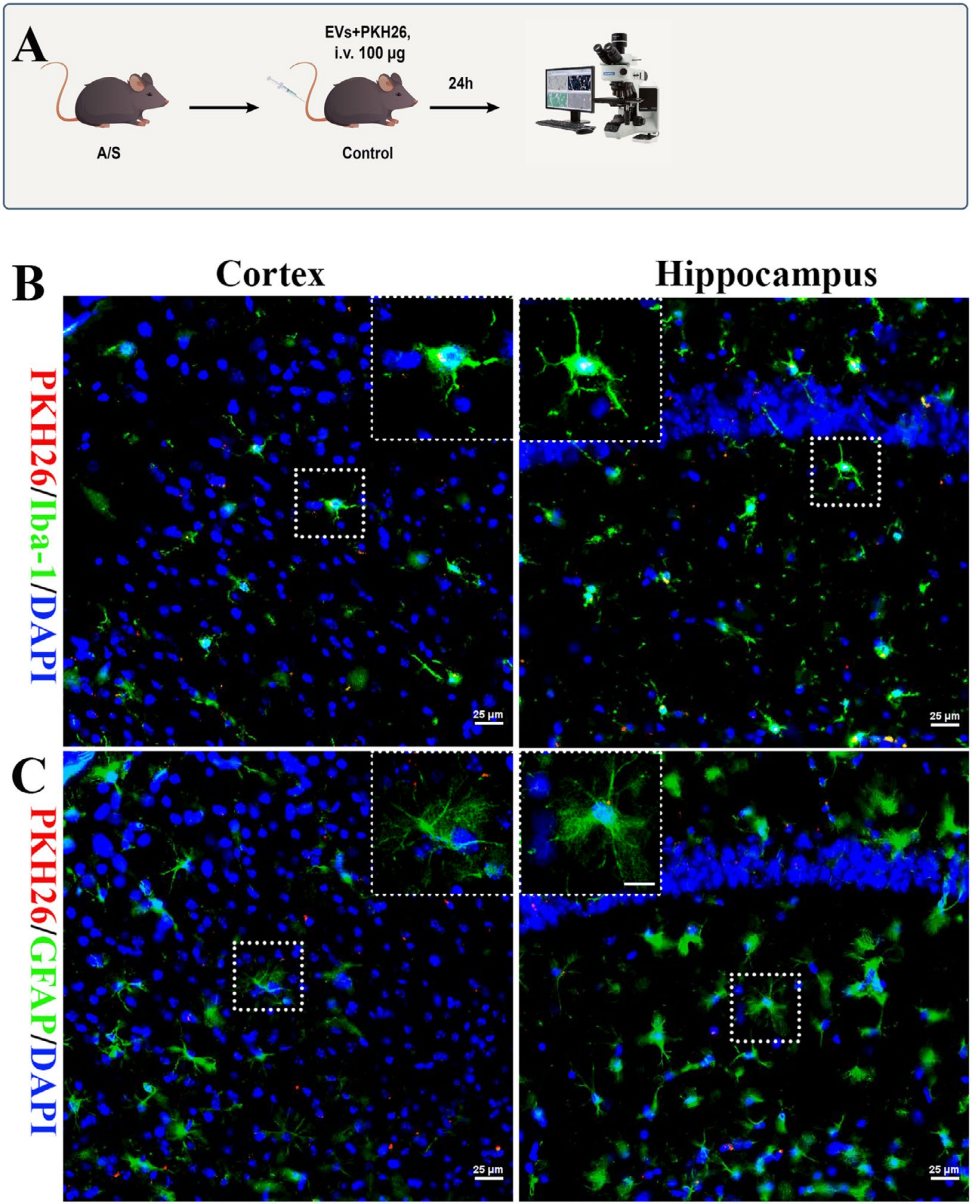
Localization of circulating EVs in the mouse brain after injection into the tail vein. (A) Circulating EVs treatment protocol. (B) Representative fluorescence images reveal the co‐localization of labeled EVs with microglia in the cortical and hippocampal areas. EVs (red), Iba‐1 staining to detect microglia (green), and nuclei staining with DAPI (blue). (C) Representative fluorescence images reveal co‐localization of labeled EVs with astrocytes in the cortex and hippocampus. EVs (red), GFAP staining to detect astrocytes (green), and nuclei stained with DAPI (blue). Scale bar: 25 μm.

To determine whether circulating EVs could enter the brain directly from the bloodstream, we used lipophilic PKH26 dye to label the EVs. Subsequently, we intravenously administered labeled circulating EVs to aged C57BL/6J mice 24 h after the intravenous injection. A freshly frozen mouse brain section was observed using fluorescence microscopy (Figure 2A). To determine the labeled EVs‐positive cell phenotype, immunofluorescence staining of the brain sections was performed using antibodies against GFAP (for astrocytes) and Iba‐1 (for microglia). Cells that exhibited positive labeling for EVs were detected in the cerebral cortex and the hippocampus. These cells demonstrated co‐localization with Iba‐1 (Figure 2B), whereas no co‐localization with GFAP was observed (Figure 2C). These results suggest that intravenously administered serum exosomes can cross the BBB and are primarily internalized by microglia.

### 3.3 Anesthesia and Surgery‐Derived Circulating EVs Induced Delirium‐Like Behavioral Changes in Recipient Mice

**FIGURE 3:**
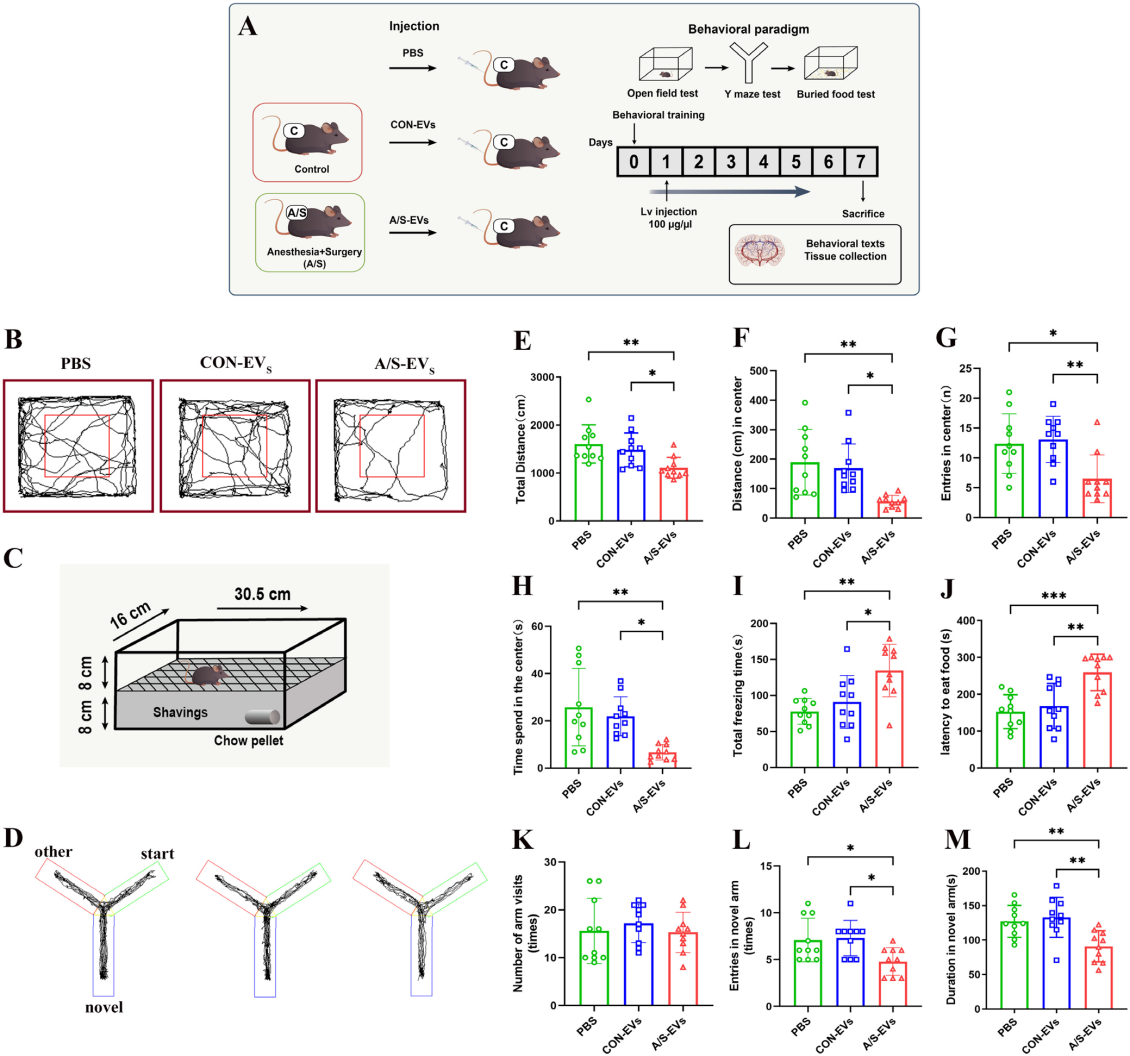
Results of behavioral analysis after tail vein injection of circulating EVs. (A) Schematic representation of experimental design. (B) Movement trajectory of mice in the open field test. (C) Schematic representation of the buried food test. (D) Movement trajectories of mice in the Y‐maze experiments. (E–I) Behavior analysis of open field test, including total distance (E), distance to the center (F), number of trips to the center (G), time spent in the center (H), and total freezing time (I). (J) Latency for mice to find food. (K–M) Behavioral analysis of the Y‐maze test, including the total number of arm entries (K), total number of arm entries (L), and duration in new arms (M). Data are expressed as the mean ± standard deviation. *p < 0.05, **p < 0.01, ***p < 0.001 (*n* = 10).

We adopted the behavioral testing method for evaluating POD in mice proposed by Peng et al. [18] to ascertain the impact of anesthesia and surgery‐derived circulating EVs on cognitive function. The experimental procedure is illustrated in Figure 3A. The results of the open field test revealed that the A/S‐EVs group exhibited a reduced total distance, center distance, number of entries to the center, time spent in the center, and prolonged freezing time compared to PBS or CON‐EVs groups (Figure 3B,E–I). The circulating EVs of aged mice injected with anesthesia and surgery are exhibited to alter behavioral characteristics, including anxiety, agitation, caution, or timidity when responding to a new environment. The results of the buried food test revealed a prolonged latency in finding food in the A/S‐EVs group compared to PBS or CON‐EVs groups (Figure 3C,J). This exhibits an impairment in the ability of autonomous food searching in aged mice injected with circulating EVs from anesthesia and surgery, suggesting inattention, disorganized thinking, and altered levels of consciousness. In the Y‐maze test, the A/S‐EVs group demonstrated fewer entries into new arms and a shorter time spent in new arms than the PBS or CON‐EV groups (Figure 3D,K–M). This implies that circulating EVs injected into aged mice after anesthesia can induce impairments in spatial learning and memory abilities, which are associated with changes in consciousness and cognitive integrity.

### 3.4 Anesthesia and Surgery‐Derived Circulating EVs Induced Microglial Activation in the Hippocampal CA1 Area of Recipient Mice

**FIGURE 4:**
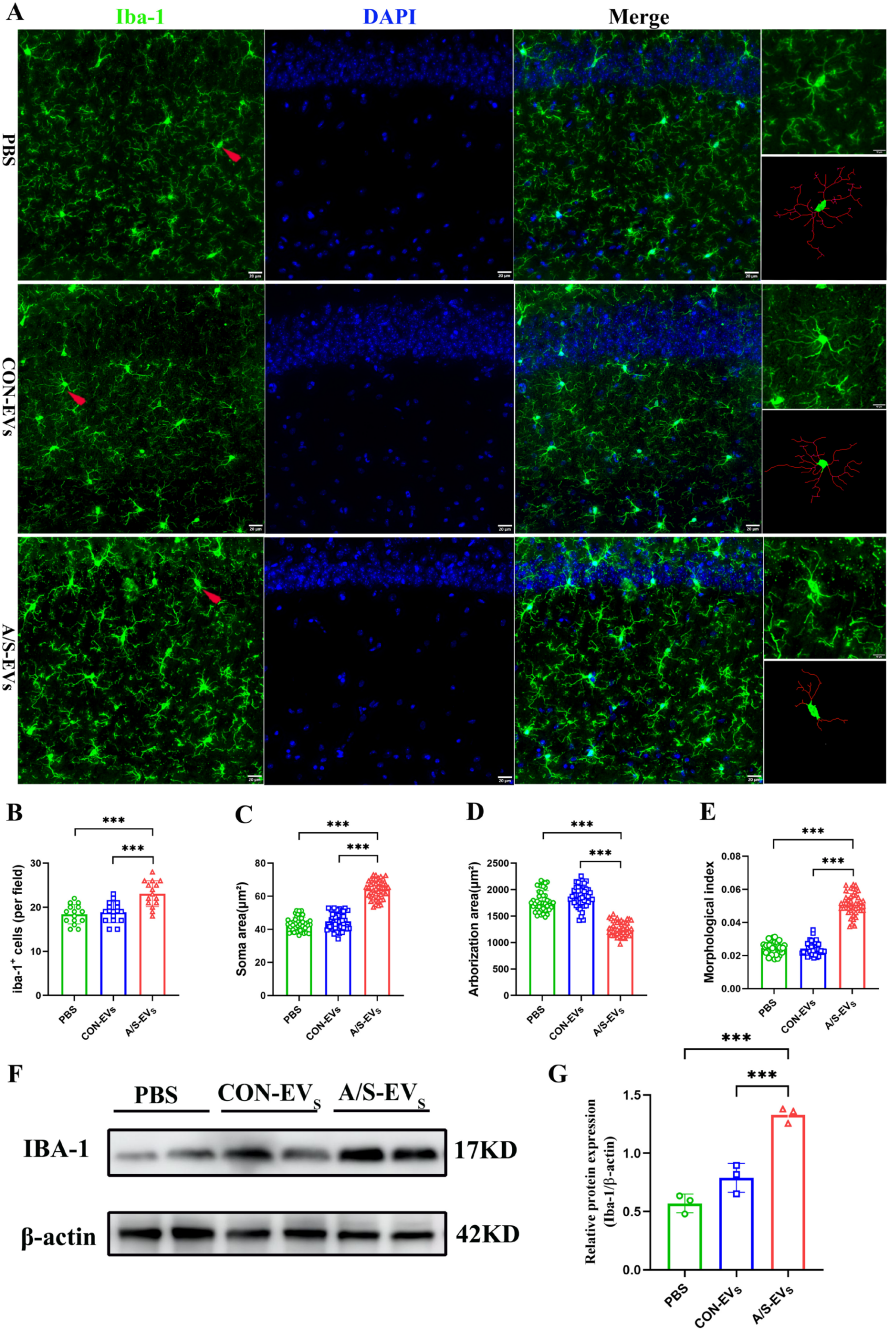
Fluorescence expression and morphological analysis of microglia in the hippocampal CA1 area. (A) Representative images of immunofluorescence of microglia in the hippocampal CA1 area of the three groups of mice, revealing Iba‐1 staining to detect microglia (green) and cell nuclei using DAPI staining (blue). Scale bar: 20 μm. The right side of the picture displays representative microglia and the corresponding microglial cytoskeletal diagram. (B) Number of Iba‐1 positive cells per field, *n* = 5 mice, 15 fields per group. ***p < 0.001. (C–E) Microglial morphological analysis, including microglia soma area (C), microglia arborization area (D), and morphological index (E), *n* = 5 mice, 15 fields, 45 cells per group. ***p < 0.001. (F) Immunoblotting of Iba‐1 in the hippocampal tissue. (G) Quantitative analysis of Iba‐1 protein expression (*n* = 3). ***p < 0.001.

Microglial activation is often characterized by an increase in cell number and changes in cell morphology, such as an increase in cell body size and retraction of projections [19]. In our study, we conducted immunofluorescent staining of brain slices using the microglia marker antibody Iba‐1 to assess the number and morphology of Iba‐1‐positive microglia in the hippocampal CA1 area of mice from different experimental groups. We observed that the number of Iba‐1‐positive microglia significantly increased in the A/S‐EVs group compared to PBS and CON‐EVs groups. Simultaneously, there was a nonsignificant difference between PBS and CON‐EVs groups (Figure 4A,B). Additionally, skeletalized imaging revealed that microglia in the A/S‐EVs group exhibited decreased branch density and increased cell volume compared to the other groups (Figure 4A, right side). The morphological analysis further revealed that microglia in the A/S‐EVs group revealed a larger soma area (Figure 4C), reduced arborization area (Figure 4D), and a higher morphological index (Figure 4E), indicating an increase in the cell soma area relative to the arborization area. In addition, we detected changes in Iba‐1 protein expression in the hippocampal tissue of the three groups of mice by Western blotting. The results demonstrated that compared with PBS and CON‐EVs groups, Iba‐1 protein expression increased in the A/S‐EVs group, with a nonsignificant difference between PBS and CON‐EVs groups (Figure 4F,G). These results indicated that circulating EVs from anesthesia surgery induced microglial activation in the hippocampal CA1 area of recipient mice.

### 3.5 Anesthesia and Surgery‐Derived Circulating EVs Increased Expression of Pro‐Inflammatory Factors in the Hippocampus of Recipient Mice

**FIGURE 5:**
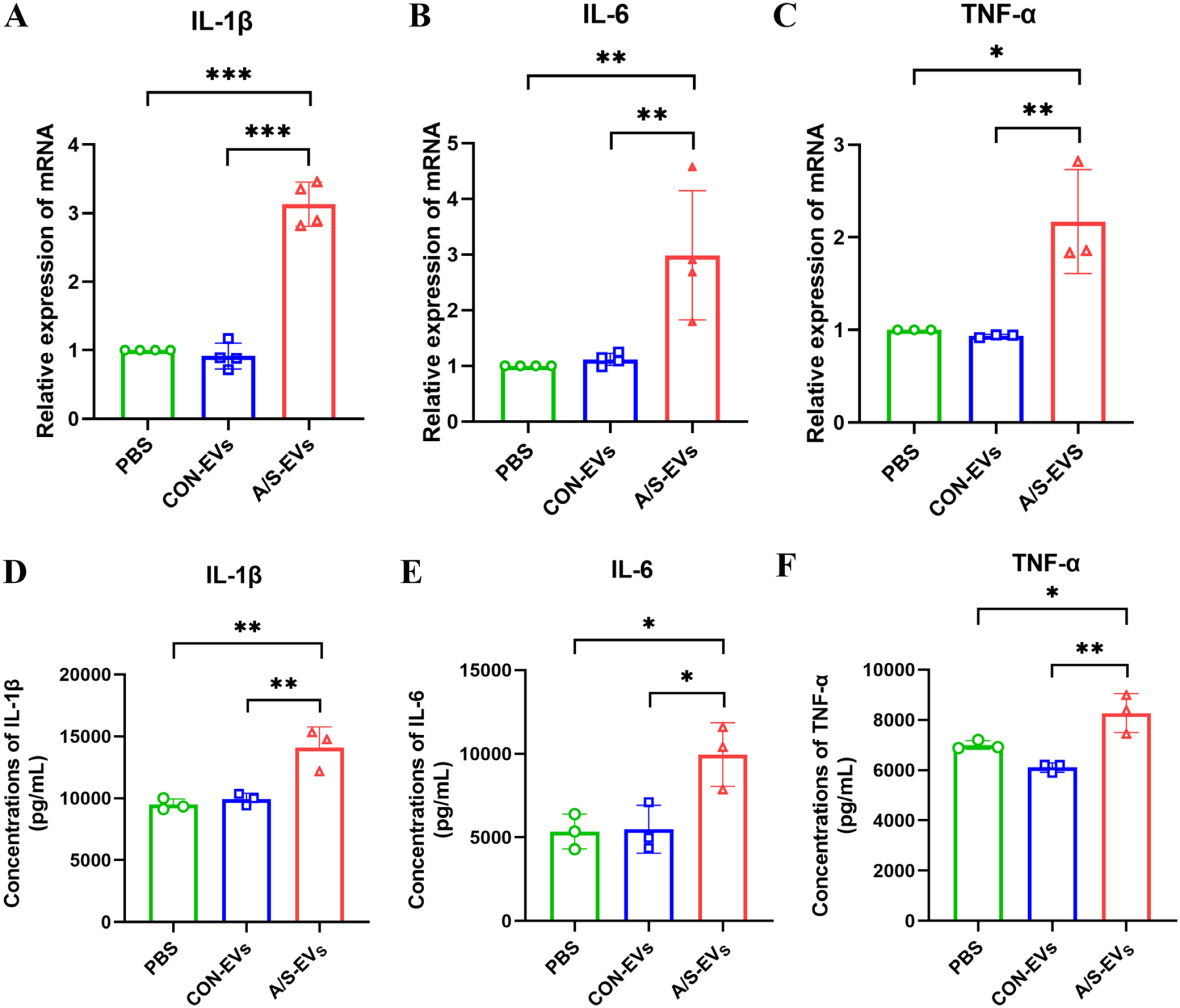
Expression levels of pro‐inflammatory factors in hippocampal tissue. (A) Relative expression levels of IL‐1β mRNA (*n* = 4). (B) Relative expression of IL‐6 mRNA (*n* = 3). (C) TNF‐α mRNA relative expression level (*n* = 3). (D) ELISA detects the relative expression level of IL‐1β in hippocampal tissue (*n* = 3). (E) ELISA detects the relative expression level of IL‐6 in hippocampal tissue (*n* = 3). (F) ELISA detects the relative expression of TNF‐α in hippocampal tissue (*n* = 3). *p < 0.05, **p < 0.01,***p < 0.001.

Microglial activation can promote the release of inflammatory factors. Therefore, we extracted hippocampal tissue from mice and used PCR and ELISA to detect changes in the expression of pro‐inflammatory factors associated with microglial activation. The results revealed that compared with PBS and CON‐EVs groups, the content of interleukin (IL)‐1β, IL‐6, and TNF‐α in the hippocampal tissue of recipient mice, and their mRNA expression levels all increased in the A/S‐EVs group, with the nonsignificant difference between PBS and CON‐EVs groups (Figure 5).

### 3.6 Dendritic Spine Deletion in the CA1 Area of the Hippocampus of Recipient Mice Was Triggered by Anesthesia and Surgery‐Derived Circulating EVs

**FIGURE 6:**
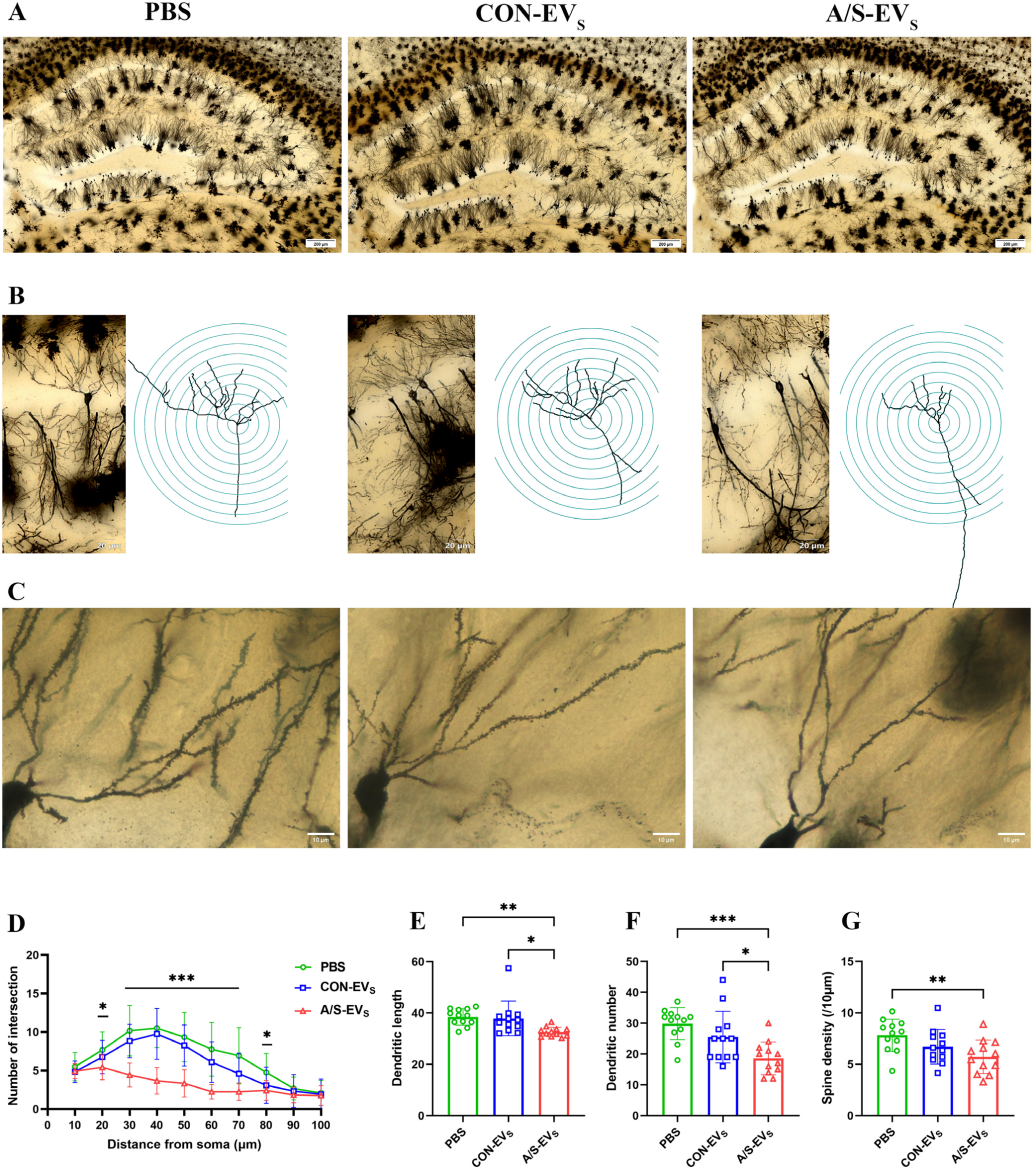
Effects of circulating EVs on dendritic spine density and morphology in the CA1 region of the mice hippocampus. (A) Representative hippocampal Golgi‐Cox staining images. Scale bar: 200 μm. (B) Dendritic branches of CA1 pyramidal neurons in the hippocampus and Sholl's analysis of the dendritic complexity of pyramidal neurons. Scale bar: 20 μm. (C) Representative spine micrographs of secondary dendrites of hippocampal CA1 pyramidal neurons. Scale bar: 10 μm. (D) Quantitative analysis of the number of dendritic branches in the hippocampal CA1 pyramidal neurons. (E) Quantitative analysis of the dendritic length in hippocampal CA1 pyramidal neurons. (F) Number of dendrites in hippocampal CA1 pyramidal neurons. (G) Spine density of dendrites in hippocampal CA1 pyramidal neurons. *p < 0.05, **p < 0.01, ***p < 0.001 (*n* = 12 per group).

To explore the potential mechanisms underlying the neurobehavioral effects of circulating EVs, dendritic spine density and morphological changes were observed in the CA1 region of the mouse hippocampus, a crucial anatomical region involved in memory formation and learning. Compared to PBS and CON‐EV_S_ groups, A/S‐EVs group revealed a reduced number of dendritic branches in the CA1 region of the brain, with more pronounced effects observed in the region 30–70 μm away from the cell body (Figure 6A,B,D), with shorter dendritic lengths (Figure 6A,C,E), fewer dendritic number (Figure 6A,C,F), and the low density of the CA1 spines (Figure 6A,C,G). The findings indicated that administering anesthesia and surgery‐derived circulating EVs removed dendritic spine structures in normal‐aged animals.

### 3.7 Anesthesia and Surgery‐Derived Circulating EVs Damaged the Ultrastructure of Neurons and Synapses in the CA1 Area of Recipient Mice

**FIGURE 7:**
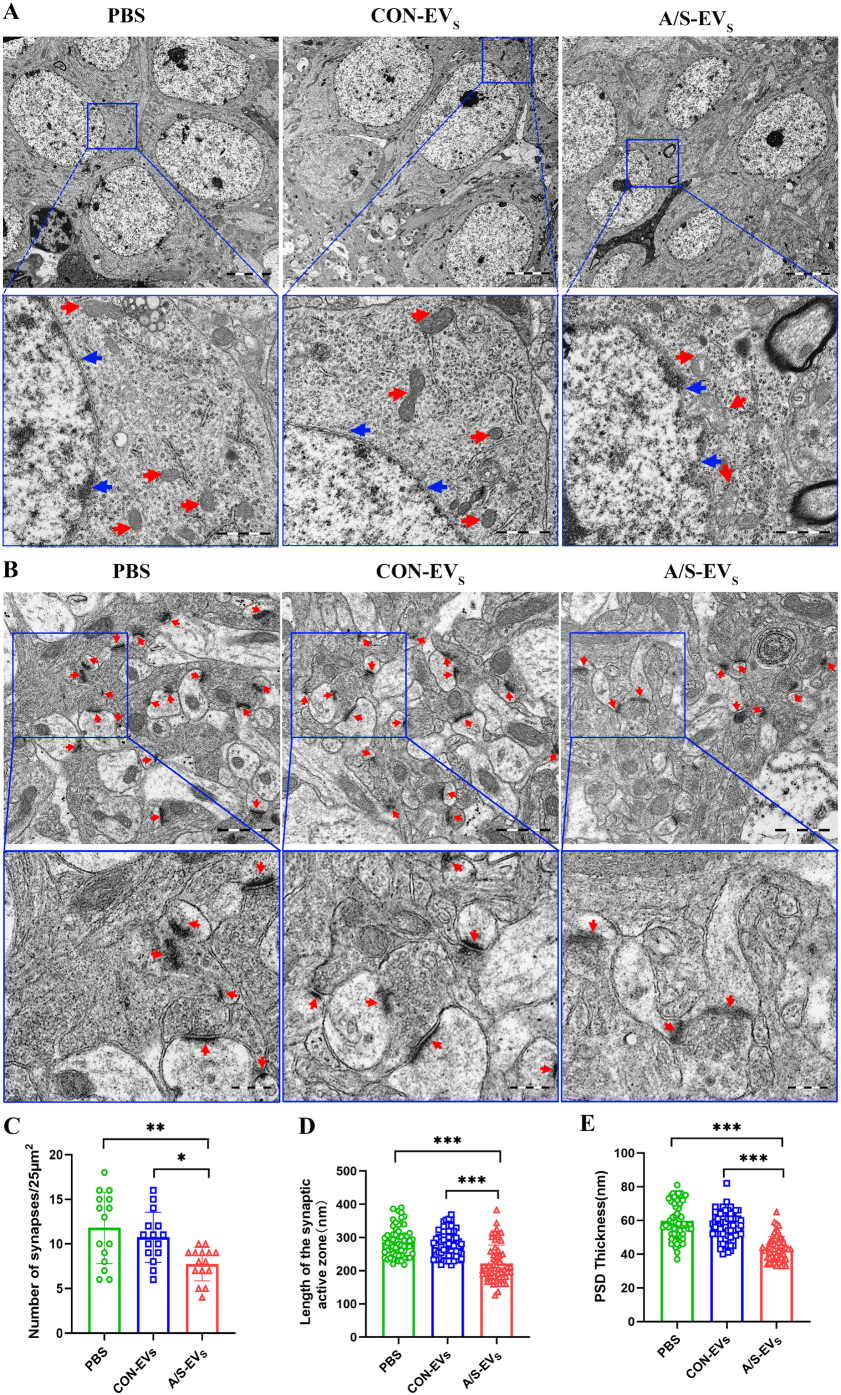
Ultrastructure and analysis of neurons and synapses in CA1 region of mouse hippocampus. (A) Representative diagram of pyramidal neuron morphology in the hippocampal CA1 area; blue arrow indicates the nuclear membrane; red arrow indicates the mitochondria. Scale bar: 1 μm. (B) A representative image displays the synaptic ultrastructure, with a red arrow indicating the synapse. Scale bar: 500 nm. (C) Histogram illustrating the number of synapses per 25 μm^2^ in each group. (D) Quantitative analysis of the length of the synaptic active zones in each group. (E) Quantitative analysis of the PSD thickness in each group. *p < 0.05, **p < 0.01, ***p < 0.001 (*n* = 3 mice per group, 15 fields, 60 synapses in each group).

Microglial activation influences the structure and function of neurons and synapses, and cognitive dysfunction is often related to synaptic damage [20]. To investigate this, we used TEM to examine the neurons and synapses ultrastructure in the hippocampal CA1 area. Our observations revealed that neuronal cells in the hippocampal CA1 area of PBS and CON‐EV_S_ groups exhibited normal morphology, smooth and intact nuclear membranes, and normal mitochondrial morphology in the cytoplasm. Contrarily, neuronal cells in the A/S‐EVs group revealed shrunken nuclear membranes with invaginations, as well as vacuoles and swelling in the mitochondria (Figure 7A). Analysis of the synapse ultrastructure indicated that the A/S‐EVs group had sparser synapses with blurred outlines and reduced numbers compared to PBS and CON‐EV_S_ groups (Figure 7B,C).

Additionally, the length of the synaptic active zone was shorter (Figure 7B,D), and the postsynaptic density (PSD) was thinner (Figure 7B,E) in the A/S‐EVs group. Nonsignificant differences were observed between PBS and CON‐EV_S_ groups. These findings suggest that circulating EVs from anesthesia and surgery in mice can induce damage to hippocampal CA1 neuron cells in recipient mice, decreasing synaptic number, shortening the length of the synaptic active zone, and thinning the PSD.

### 3.8 Anesthesia and Surgery Modified the Circulating EVs Content in Aged Mice

Circulating EVs from mice undergoing anesthesia and surgery induced cognitive behavioral abnormalities and pathological changes in brain nerve cells in recipient mice. Contrarily, circulating EVs from the control mice did not exhibit the same pathological and behavioral effects. We hypothesized that proteins and miRNAs present in circulating EVs may play a role in mediating these effects. Consequently, we performed proteomic and miRNA sequencing analyses of circulating EVs to identify potential pathogenic molecules.

#### 3.8.1 Effect of Anesthesia and Surgery on the Circulating EVs Proteome

We analyzed circulating EVs protein profiles in aged mice after 24 h in the A/S and CON groups using untagged proteomics. In total, 6365 peptides and 788 proteins were identified. Principal component analysis (PCA) of proteomic data revealed a clear separation between A/S and CON groups, indicating significant differences in protein expression profiles between the two groups. In contrast, the protein expression within each group was relatively consistent (Figure S1A). Protein quantification analysis revealed 98 differentially expressed proteins (fold change > 2, *p* < 0.05) when comparing A/S and CON groups. Among these, 39 proteins were upregulated, and 59 were downregulated. The differentially expressed proteins were exhibited in a volcano plot according to their folding changes and *p* values (Figure S1B). A clustered heatmap of the two clusters was constructed using normalized data to outline the distribution of expressed EV proteins (Figure S1C). The subcellular localization analysis of differentially expressed proteins (Figure S1D) exhibited that they were mostly found in the cytoplasm and extracellular matrix, which fits with the characteristics of EVs. To gain an insight into the potential biological functions of differentially expressed proteins, we applied the Reactome, GO, and KEGG pathway analysis (Figure S1E). Reactome analysis revealed associations between platelet degranulation, neutrophil degranulation, and extracellular matrix organization. GO analysis highlighted their involvement in acute‐phase responses, protein–lipid complex remodeling, negative regulation of hydrolase activity, innate immune response, humoral immune response, bacterial defense response, biological processes related to symbiotic interactions, and extracellular matrix organization. KEGG pathway analysis was performed on the differential proteins screened, and it was observed that these proteins were mainly concentrated in the complementation and coagulation cascade pathways.

#### 3.8.2 Effect of Anesthesia and Surgery on Circulating EVs miRNAs

To conduct a more comprehensive examination of the impact of anesthesia and surgery on EV content, we performed miRNA sequencing. First, a sample cluster analysis with DESeq2 homogenization of mature small RNA expression was performed to ensure the reliability and rationality of the experiments (Figure S2A). A volcano plot presents the overall distribution of the EVS miRNAs identified in this study (Figure S2B). Gray dots represent miRNAs without significant changes, and red dots signify miRNAs with significant differences (*p* < 0.05, fold change > 2.0; Figure S2B). We identified 90 significantly differentially expressed miRNAs, with 32 upregulated genes (to the right of the second vertical dashed line on the *y* axis) and 58 downregulated genes (to the left of the first vertical dashed line on the *y* axis). We generated a heat map of 90 differentially expressed miRNAs to illustrate distinguishable miRNA expression profiles in the samples (Figure S2C). The top 20 significantly enriched genes with enrichment scores were subjected to GO functional analysis to annotate and hypothesize the functions of the miRNAs (Figure S2D). Differentially expressed miRNAs were significantly associated with the following terms: Wnt signaling pathway, transcriptional coactivator activity, synaptic membrane, synaptic tissue, small GTPase‐mediated signaling, neurogenesis regulation, regionalization, protein localization to the cell periphery, positive regulation of cell projection organization, pattern specification process, neuron‐to‐neuron synapse, axon genesis, forebrain development, distal axon, cell–cell signaling pathway, cell‐leading edge, cell structure assembly, and amoeboid‐type cell migration. A KEGG pathway map was constructed to identify the top 20 significantly enriched pathways (Figure S2E). This includes pathways related to CNS diseases, including Wnt signaling, Rap1 signaling, PI3K‐AKT signaling, mTOR signaling, MAPK signaling, Hippo signaling pathways, and axon guidance. Reactome pathway enrichment analysis predicted the functional roles and interactions of miRNA changes in circulating EVs after anesthesia and surgery within biological processes (Figure S2F). This includes major pathways, including the VEGFA‐VEGFR2 pathway, transmission through chemical synapses, SUMOylation, SUMO E3 ligases, SUMOylate target proteins, signaling by receptor tyrosine kinases, RHO GTPase cycle, nervous system development, and axon guidance.

#### 3.8.3 Candidate Target Protein SAA1 Verification

**FIGURE 8:**
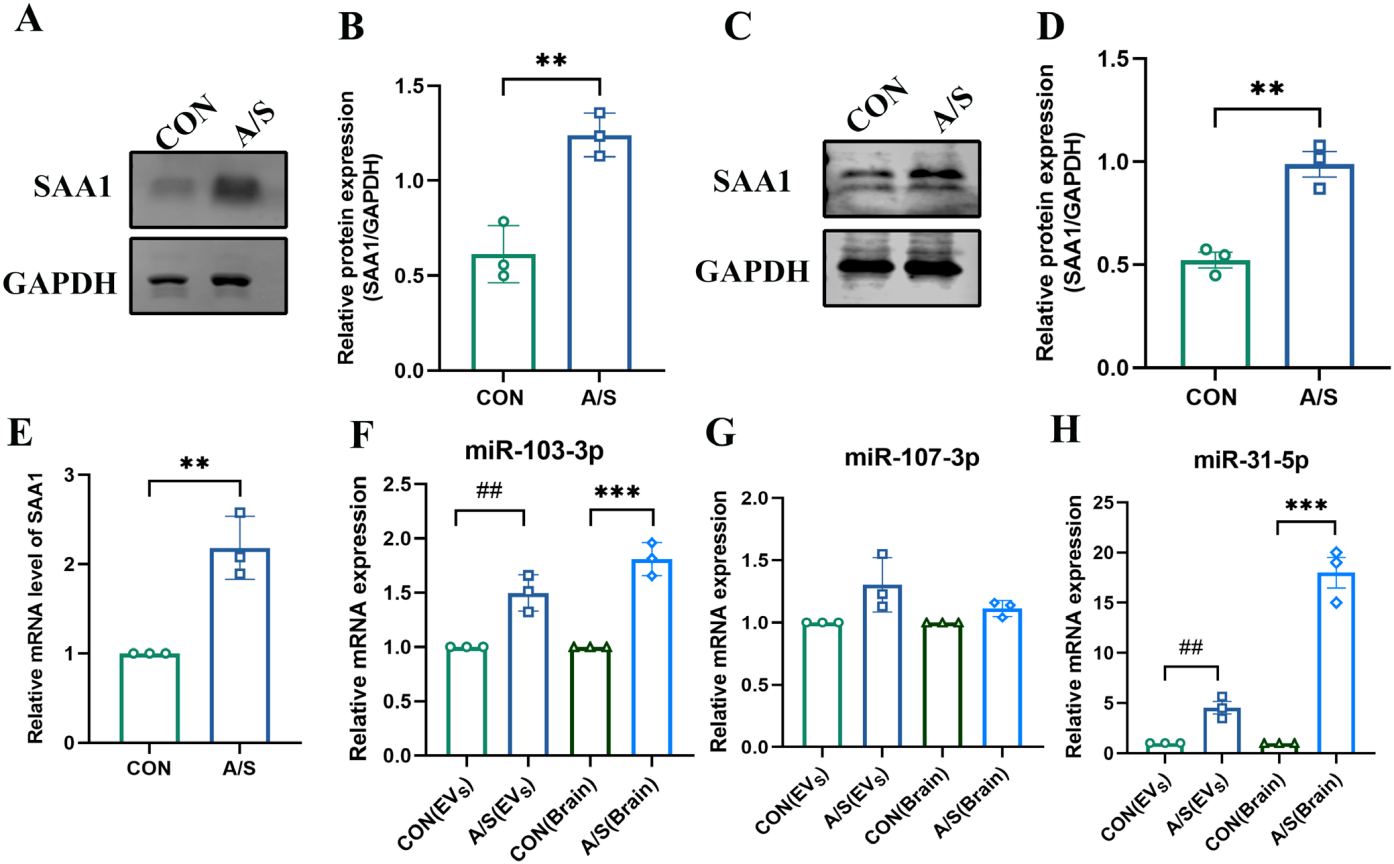
SAA1 and candidate miRNA expression levels in serum and hippocampal tissues. (A) The expression of SAA1 protein in serum of the CON group and A/S group was detected by Western blotting. (B) Quantitative analysis of SAA1 protein expression in serum of the CON and A/S groups. (C) The expression of SAA1 protein in hippocampal tissue of the CON group and A/S group was detected by Western blotting. (D) Quantitative analysis of SAA1 protein expression in hippocampal tissue of the CON and A/S groups. (E) The mRNA expression and analysis of SAA1 in the CON group and A/S groups. (F) Relative expression levels of miR‐103‐3p expression in circulating EVs and hippocampal tissue (CON vs. A/S groups). (G) Relative expression levels of miR‐107‐3p in circulating EVs and hippocampal tissue (CON vs. A/S groups). (H) Relative expression levels of miR‐31‐5p in circulating EVs and hippocampal tissue (CON vs. A/S groups). **p < 0.01, ***p < 0.001,^##^ *p* < 0.01, (*n* = 3).

The candidate target protein SAA1 was verified by analyzing the differential proteins and their biological functions, followed by a literature review to identify relevant proteins. SAA1 was selected as a candidate target protein. Western blotting and RT‐PCR were used to assess the changes in SAA1 total protein and mRNA expression in the serum and brain hippocampal tissue of A/S and CON groups. The results indicated elevated levels of SAA1 total protein in the serum and hippocampal tissue of mice in the A/S group compared to the CON group and a significant increase in mRNA expression of SAA1 in the hippocampal tissue (Figure 8). These findings suggest that SAA1 may be a target protein in POD development.

#### 3.8.4 Candidate miRNA Validation

Combining differential miRNA biological analysis and literature reports, candidate miRNAs are related to neuroinflammation‐related diseases. Among the significantly changed miRNAs, miR‐103‐3p, miR‐107‐3p, and miR‐31‐5p were verified as candidate miRNAs. RT‐PCR was used to analyze the expression levels of three miRNAs (miR‐103‐3p, miR‐107‐3p, and miR‐31‐5p) in the circulating EVs and hippocampal tissues of the CON and A/S groups. The results revealed that compared with the CON group, the expression levels of miR‐103‐3p and miR‐31‐5p in circulating EVs and hippocampal tissue of the A/S group significantly increased. However, miR‐107‐3p levels did not change significantly in circulating EVs and hippocampal tissue of both groups (Figure 8).

#### 3.8.5 Overexpression of miR‐103‐3p Significantly Reduces the Viability of Microglial Cells and Promotes Secretion of Proinflammatory Factors

To determine the regulation of miR‐103‐3p, BV2 cells were transfected with fluorescein amidite (FAM)‐labeled small interfering RNA (siRNA) for 48 h. miR‐103‐3p was effectively overexpressed in BV2 cells via transfection (Figure S3A,B). Overexpression of miR‐103‐3p significantly reduced the viability of BV2 cells, as measured by CCK‐8 assay (Figure S3C). Immunofluorescence analysis further demonstrated that the number of CD86‐positive BV2 cells was increased (Figure S3D,E). Consistently, RT‐PCR analysis revealed elevated mRNA expression levels of pro‐inflammatory factors (IL‐1β, TNF‐α, and IL‐6) (Figure S3F). These results indicate that miR‐103‐3p overexpression promotes the expansion of CD86‐positive BV2 cells and upregulates the expression of pro‐inflammatory factors mRNAs.

## 4 Discussion

POD is a form of cognitive impairment that manifests acutely and exhibits certain temporal characteristics. It often occurs within 24–72 h after anesthesia and surgery, in which aging is an independent risk factor for its occurrence [21]. A recent study reported [14] that surgical trauma modified the serum EVs content in the peripheral circulation of mice, which was characterized by notable variations in the protein and miRNA expression. These molecules commenced 6 h after surgery, peaked at 24 h, and declined gradually after 72 h. This is consistent with the timing of structural, metabolic, functional, and behavioral changes in the brain observed after peripheral surgery in mice [22]. Consequently, to construct an animal model for this study, we selected aged male mice and performed laparotomy under sevoflurane anesthesia. This method is commonly used in postoperative delirium research and is frequently used in animal experiments investigating POD [23]. Subsequently, we used the ExoQuick kit to isolate EVs from the peripheral blood serum of mice 24 h after anesthesia and surgery, as well as from control aged mice.

Crossing the BBB is crucial for the peripheral systems to communicate with the brain. Previous studies have confirmed that circulating EVs can interact between the brain and circulatory system and transmit peripheral information to the brain [24]. To determine whether circulating EVs can cross the BBB and be internalized by which type of glial cells in the brain, we labeled circulating EVs with the lipophilic dye PKH26. We observed the distribution and expression of circulating EVs in the brain after 24 h by fluorescence staining. The results revealed that the labeled circulating EVs were expressed in the cortex and hippocampus (red) and co‐localized with Iba‐1 but not with GFAP, suggesting that circulating EVs can enter the brain and be internalized by microglia in the brain. Our study aligns with previous research on EVs biodistribution, in which most intravenously injected fluorescently labeled or radiolabeled EVs aggregated in less than 1 h in the liver, spleen, and lungs, whereas they were barely detectable in the brain [25]. Another study used an in vivo nanoparticle imaging system and revealed that 24 h after intravenous injection, the brain accumulated detectable fluorescently labeled EVs around the third ventricle and lateral ventricle regions, but to a much lower extent compared to the liver, spleen, and lungs [26]. EVs derived from serum may be heterogeneous in size and origin, and EVs from different cell types may have different tissue‐specific homing effects [12]. Recent studies have suggested the potential involvement of EVs in the transfer of harmful effects from the peripheral circulation to the brain, particularly concerning chronic inflammation.

Protein aggregation, dopaminergic neuron degeneration, microglial activation, and motor impairments are observed in mice after intravenous injection of Parkinson's disease serum exosomes [27]. Administering serum EVs derived from a mouse model of sepsis produced by lipopolysaccharide (LPS) enhanced the microglial cell activation, the proliferation of astrocytes, and elevated production of IL‐6 and TNF‐α in the hippocampus and cerebral cortex of healthy mice [12]. The injection of EVs from the peripheral blood of aged mice into young mice resulted in modifying gene expression within the brains of young animals [28]. After LPS injection or partial hepatectomy, peripheral blood‐derived EVs could induce pro‐inflammatory cytokine secretion by the neuroglial cell system [1, 29]. The authors suggested that the EVs‐related pro‐inflammatory signaling pathways contribute to neuroinflammatory response induction. In this study, after injecting circulating EVs from mice after anesthesia surgery into control mice, recipient mice exhibited delirium‐like behavioral changes, microglial activation in the hippocampal CA1 area, increased levels of pro‐inflammatory factors TNF‐α, IL‐β, and IL‐6 in the hippocampal tissue, and damage to neurons and synapses. These pathological changes are consistent with the pathological alterations associated with postoperative delirium caused by anesthesia and surgery. The pathological basis of perioperative neurocognitive disorders is the neuroinflammatory response, microglial activation, and damage to synaptic plasticity [30]. Contrarily, aged mice treated with circulating EVs from control aged mice did not display the previously stated pathogenic and behavioral alterations. This outcome may be due to anesthesia and surgery, which modify the content and biological properties of circulating EV_S_. EVs are rich in transmembrane proteins, cell adhesion molecules, scaffolding proteins, RNA‐binding proteins, RNA, DNA, complex glycans, and other substances. However, distinct EVs populations have varying enrichments of specific compounds [31]. Variations in EVs content elicit distinct responses in the receptor cells [32]. Consequently, high‐throughput techniques were used to assess the impact of anesthesia and surgery on the protein and miRNA content of circulating EVs. Proteomic analysis revealed that the proteins that exhibited increased expression in circulating EVs after anesthesia and surgery may be primarily classified into three categories: Proteins related to muscle function and structure (PYGm, Ttn, and FLNC), proteins involved in acute phase stress response (SAA1, PTX3, A2M, and Serpina3n), and proteins related to lipid metabolism regulation (APOB and APOA5). Based on previous studies, these increased proteins are associated with muscle function and structure, which are linked to muscle injury caused by surgical trauma. Administering anesthesia and surgical trauma elicits an inflammatory response, leading to a prompt protein upregulation involved in the stress acute phase response within a few hours [33]. These proteins, SAA1, PTX3, A2M, Serpina3n, APOB, and APOA5, regulate various physiological and pathological processes, including, but not limited to, the inflammatory response, immune response, tissue repair, and lipid metabolism [34, 35, 36, 37]. SAA1 is an acute phase response protein mainly secreted by the liver and is also a “danger signal” in the inflammatory process [38]. Studies have revealed elevated serum SAA protein levels are associated with neuroinflammation and neurodegenerative responses [39]. SAA1 can cross the BBB, promote the aggregation of brain Aβ protein, and induce an increase in the number of microglia in the brain and pro‐inflammatory factor expression levels, leading to depression‐like behavioral changes in mice [40]. In the case of abundant Aβ protein in the brain, SAA1 can accelerate neuronal damage, exacerbate neuroinflammatory responses, and further lead to memory function decline [41]. Some studies have reported that SAA1 can activate NOD‐like receptor thermal protein domain‐associated protein 3 in microglia within the brain, leading to the release of IL‐1 and subsequent promotion of neuroinflammatory responses following a stroke [42]. SAA1 is a potential biological marker for early detection and prognosis assessment in individuals who have experienced a stroke [42]. The changes in SAA1 total protein and mRNA expression were validated in the peripheral blood and hippocampal tissue of the A/S and CON groups using Western blotting and RT‐PCR. The results demonstrated a significant increase in SAA1 total protein and mRNA expression in the peripheral blood and hippocampal tissue of mice in the A/S group compared to the CON group. Accordingly, we hypothesized that SAA1 could be a potential target protein for POD and might serve as a novel biological marker for POD. Further comprehensive research is required to elucidate the specific role of SAA1 in POD development.

The protein fraction of EVs payloads offers valuable insights into the EVs tissue source. However, it is crucial to acknowledge the significance of miRNAs in facilitating post‐transcriptional regulation, as they play a pivotal role in comprehending and predicting phenotypic alterations in recipient cells and tissues [43]. Sequencing analysis of miRNAs in circulating EVs revealed that there were 32 miRNAs significantly upregulated (miR‐103‐3p, miR‐130a‐3p, miR‐107‐3p, miR‐378a‐3p, and miRNA‐31‐5p) in the anesthesia and surgery groups compared to the control group, and 58 miRNAs were significantly downregulated (miR‐7072‐5p, miR‐664‐3p, miR‐483‐5p, mmu‐miR‐342‐5p, and miR‐124‐3p). Notably, inhibiting miRNA‐31‐5p expression inactivates the MyD88‐NF‐κB pathway, maintains endothelial function, and reduces subarachnoid‐induced BBB disruption, neuronal apoptosis, and microglial cell inflammation [44]. The attenuation of LPS‐induced depressive‐anxiety‐like behavior and the inhibition of microglial activation and pro‐inflammatory factor release in mice can be achieved by inhibiting miRNA‐107‐3P production. As a result, miR‐107‐3p may provide a novel strategy for treating CNS disorders [45]. miR‐103‐3p modulates neural stem cell proliferation through Wnt signaling inhibition and enhances stem cell apoptosis [46]. In a stroke animal model, elevated miR‐103‐3p expression in the brain was observed, and miR‐103‐3p suppression led to decreased neuronal apoptosis and enhanced recovery of neurological function post‐stroke [47]. To investigate the differential expression of miRNAs during anesthesia and surgery, we analyzed the expression levels of miR‐31‐5p, miR‐107‐3p, and miR‐103‐3p in circulating EVs and hippocampal tissue of A/S and CON groups using RT‐PCR. Our findings indicated that compared to the CON group, the A/S group exhibited significant increases in the expression levels of miR‐31‐5p and miR‐103‐3p in circulating EVs and hippocampal tissue. Among the miRNAs hypothesized as potential targets for postoperative delirium (POD), miR‐31‐5p and miR‐103‐3p emerged as prioritized candidates. Specifically, overexpression of miR‐103‐3p was found to not only significantly reduce the viability of microglial cells but also promote the secretion of pro‐inflammatory factors, which may mechanistically contribute to neuroinflammation‐associated POD pathogenesis.

The results of target gene enrichment analyses, GO, Reactome pathway, and KEGG pathway analyses demonstrated that the target genes affected by differentially expressed miRNAs were mainly involved in regulating neurogenesis, nervous system development, synapses, and axon formation, and participating in axon guidance signaling pathways. The primary etiology of POD can be attributed to the systemic acute inflammatory response triggered by anesthesia and surgical trauma. Peripheral inflammatory substances can enter the brain through various pathways, activating central resident immune cells. This activation produces neuroinflammatory factors, ultimately affecting synaptic plasticity [48]. This implies that differential miRNAs within circulating EVs may be crucial in the neurosynaptic plasticity mechanisms underlying POD. Multiple recent investigations have also confirmed the role of peripheral blood extracellular vesicular RNA in postoperative cognitive function. There are significant differences in plasma exosomal miRNAs in sevoflurane‐induced cognitive dysfunction (postoperative cognitive dysfunction, POCD) or non‐POCD. The application of plasma exosomes derived from patients with POCD to human microglial cell line HMC3 led to a reduction in HMC3 cell survival, an increase in apoptosis, and an upregulation of pro‐inflammatory subfactors TNF‐α and IL‐1β [49]. Evidence from clinical studies has demonstrated that individuals with cognitive dysfunction exhibit abnormal expression of plasma exosomal RNAs.

Furthermore, dysregulated RNAs have been associated with the onset and progression of postoperative cognitive delirium [50]. Our study has reconfirmed that anesthesia and surgery impact the composition of circulating EVs, aligning with the previous findings reported by Mkrtchian et al. [14], with slightly different differential expressions of proteins and miRNAs. This could be due to the varying ages of the mice used and variations in anesthesia and surgical methods.

Based on the above research, we suggest that circulating EVs can transport “toxic” miRNAs and proteins that interact with neuronal cells, transport the BBB, and participate in POD. Despite the substantial findings of this study, there are still significant limitations that need to be addressed. First, this study only focused on male‐aged mice and did not investigate the impact of circulating EV_S_ derived from anesthesia and surgery on young mice. Second, the detailed molecular mechanism of signal transmission from circulating EV_S_ to neurons in the brain remains unclear. Finally, our investigations did not encompass an exhaustive exploration of the precise molecular constituents present in EV_S_, nor did we thoroughly examine the intricate methods by which these components interact with the neurological system. The mechanism by which circulating EVs contribute to POD and neurodegenerative diseases requires further investigation. Further research to mitigate or prevent the detrimental effects of circulating EVs on the nervous system is a valuable avenue needed.

## 5 Conclusions

In summary, this study indicates that anesthesia and surgery impact the composition and physiological characteristics of circulating EVs. Furthermore, the administration of anesthesia and surgery‐derived EVs to control mice led to the manifestation of delirium‐like behavioral changes and pathological changes in nerve cells in recipient mice. SAA1, miR‐103‐3p, and miR‐31‐5p are potential target molecules implicated in POD. These findings suggest that circulating EVs could serve as a novel pathway through which postoperative delirium occurs, mediating the distant effects of anesthesia and surgery on the brain.

## Author Contributions

Yubo Gao and Shaling Tang performed the experiment, collected and analyzed the data, and prepared the manuscript. Xiaoxia Yang was involved in preparing the animal models. Jun Liu contributed to behavioral testing. Hui Chen and Jiahui Li contributed to Western blot and immunohistochemical analysis. Xinli Ni contributed to the study concept and design, secured funding for the project, critically revised the manuscript. All authors reviewed the final manuscript.

## Ethics Statement

All experiments were approved by the Medical Research Ethics Review Committee of the General Hospital of Ningxia Medical University Hospital (ethics no. KYLL‐2023‐0183).

## Conflicts of Interest

The authors declare no conflicts of interest.

## Supporting information


Appendix S1.

## Data Availability

The data that support the findings of this study are available from the corresponding author upon reasonable request.
