# Supplementary material for: The Administration of Circulating Extracellular Vesicles Modified by Anesthesia and Surgery Induces Delirium‐Like Behaviors in Aged Mice
Source: CNS Neurosci Ther. 2025 Jun 19;31(6):e70483. doi: 10.1111/cns.70483 (PMC12178831; doi:10.1111/cns.70483)
Supplement: Supplementary file 1 — Appendix S1. [file CNS-31-e70483-s001.zip › cns70483-sup-0001-DataS1.pdf]

CD63 (26KD)

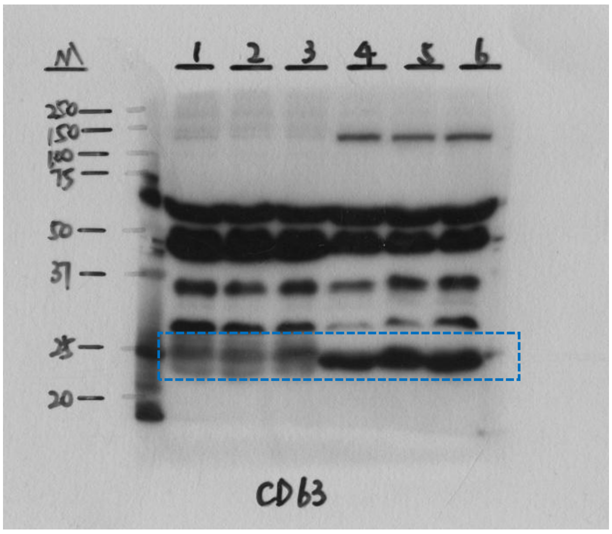

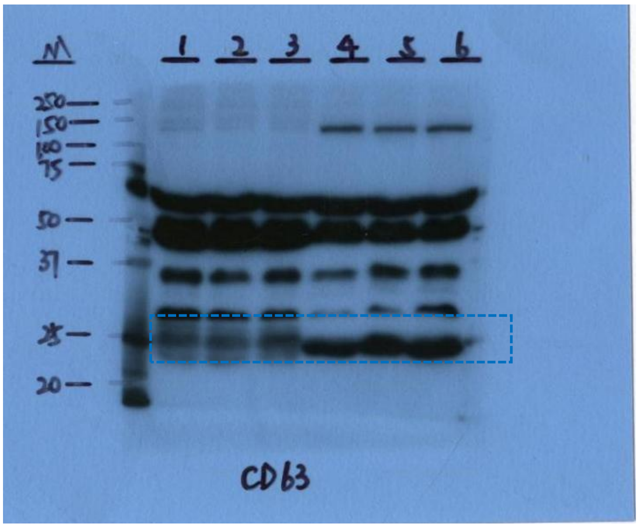

CD9 (25KD)

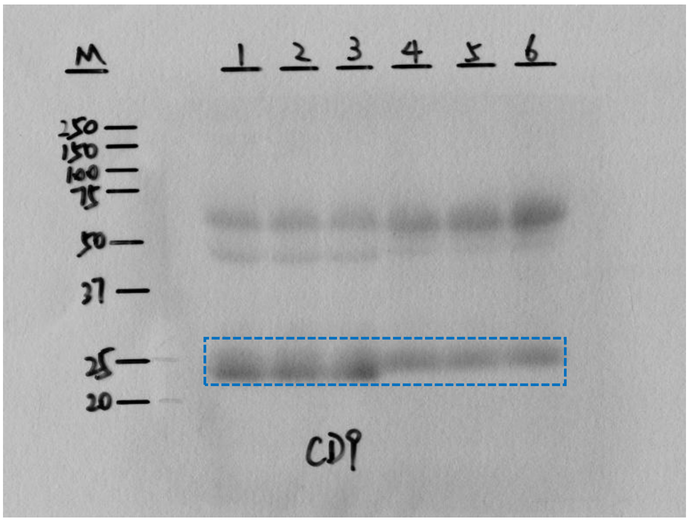

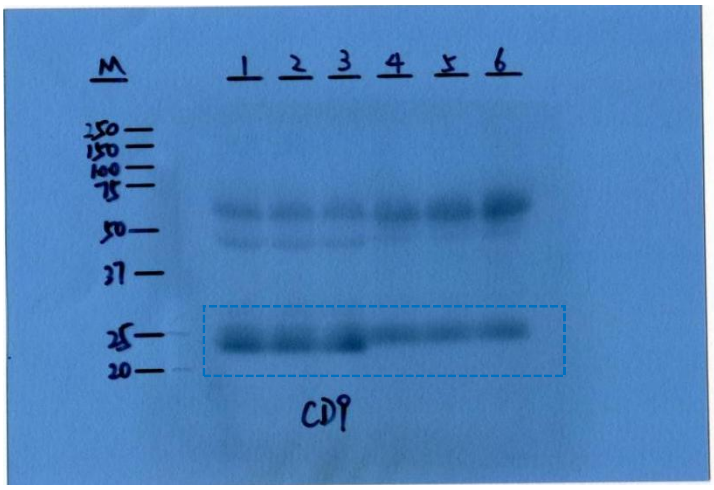

Calnexin(90KD)

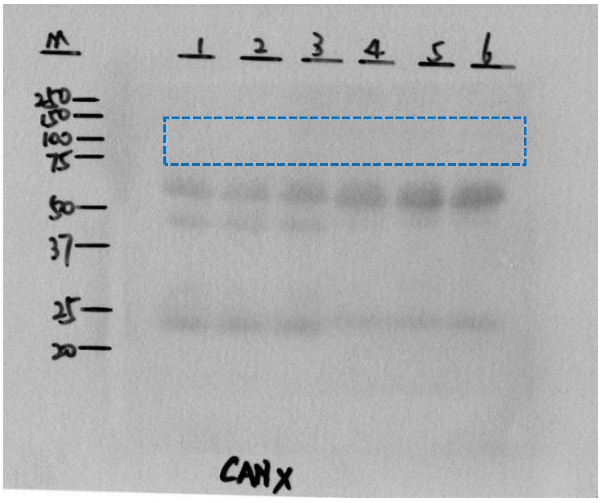

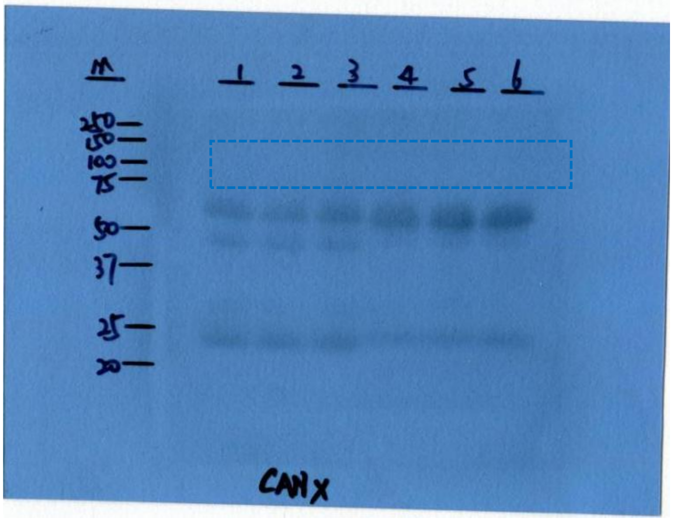

Full unedited gel/blot for Figure 1A

IBA-1

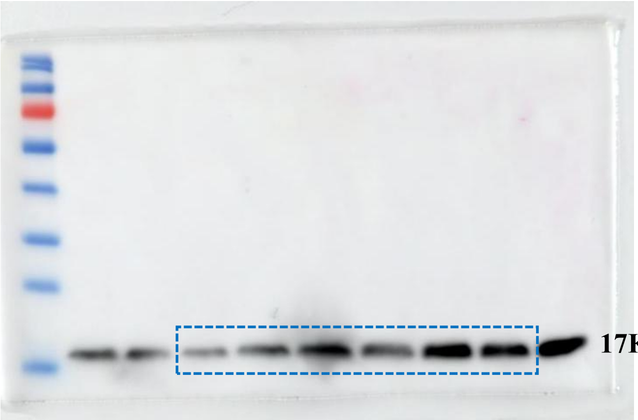

17KD

β-actin

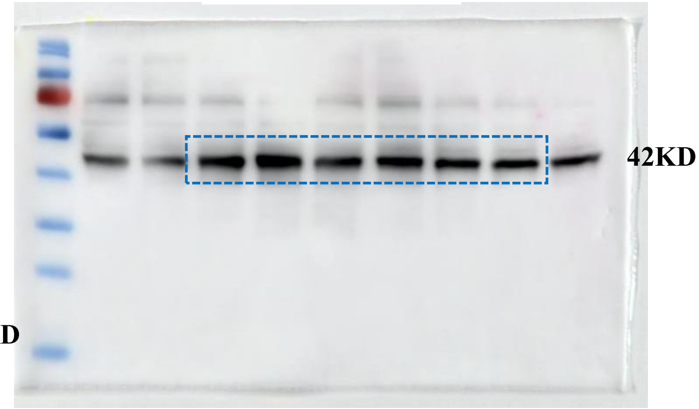

42KD

IBA-1

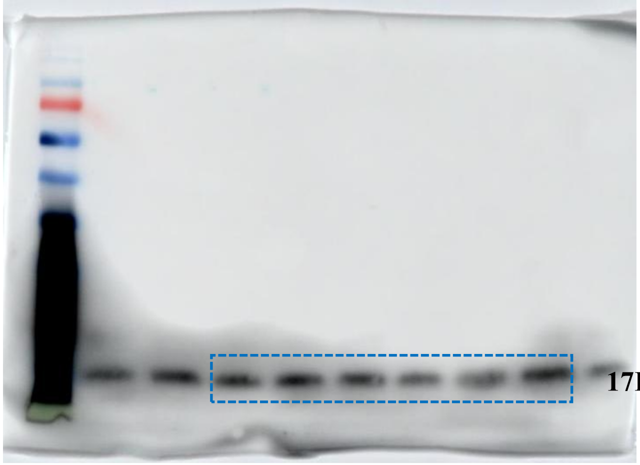

17KD

β-actin

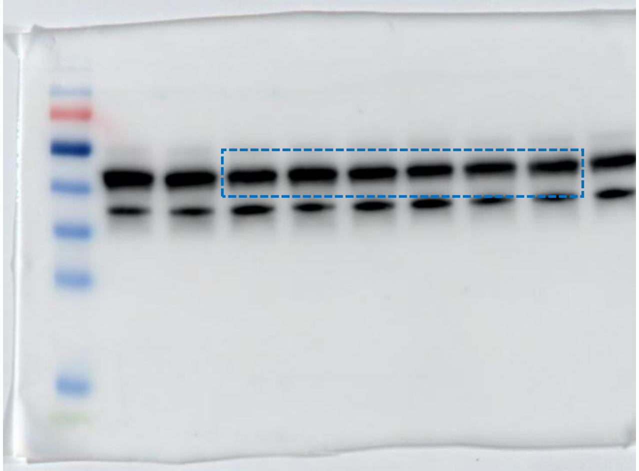

42KD

Full unedited gel/blot for Figure 4 F

SAA1 protein expression in serum

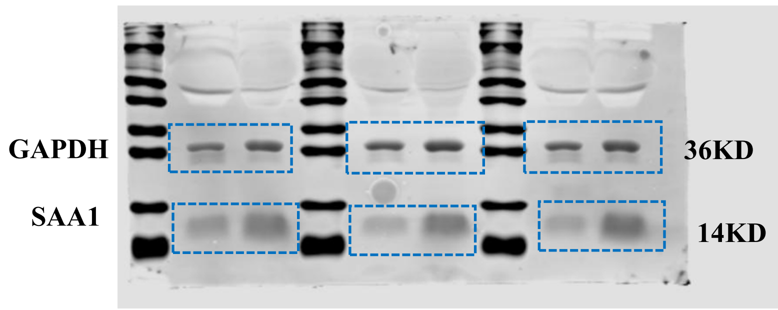

Full unedited gel/blot for Figure 8 A

SAA1 protein expression in hippocampal tissue

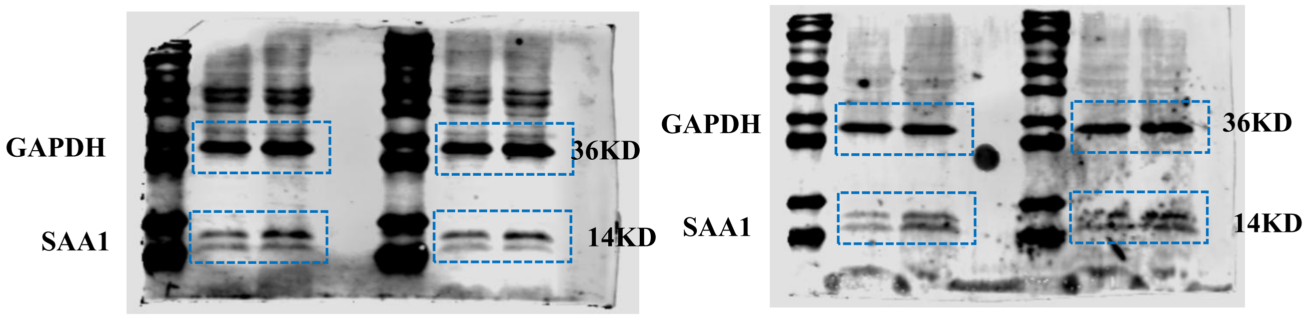

Full unedited gel/blot for Figure 8 C
