# Supplementary material for: The Administration of Circulating Extracellular Vesicles Modified by Anesthesia and Surgery Induces Delirium‐Like Behaviors in Aged Mice
Source: CNS Neurosci Ther. 2025 Jun 19;31(6):e70483. doi: 10.1111/cns.70483 (PMC12178831; doi:10.1111/cns.70483)
Supplement: Supplementary file 1 — Appendix S1. [file CNS-31-e70483-s001.zip › cns70483-sup-0002-DataS2.docx]

**Figure S3.** (A) Fluorescence imaging showing the transfection efficiency of FAM-labeled siRNA. Scale bar: 200 μm. (B) RT-PCR analysis of the relative expression level of miR-103-3p in BV2 cells after transfection with mimics-miR-103-3p. ****P* < 0.001, (n=3). (C) The viability of BV2 cells across all experimental groups was assessed using the CCK-8 assay. (D) Representative immunofluorescence images show CD86 (pro-inflammatory marker) and Iba1 (microglial marker) expression in BV2 cells under the four treatment conditions. (E) Quantitative analysis revealed a significantly increased ratio of CD86-positive cells to Iba1-positive cells in the mimics-miR-103-3p and LPS groups compared to the CON and mimics-NC groups. ****P* < 0.001 vs. CON group (n = 6). (F) RT-PCR was performed to evaluate the expression levels of pro-inflammatory factors (e.g., IL-1β, IL-6, TNF-α) in the four experimental groups.**P* <0.05, ***P* <0.01,****P*<0.001,（n=3）.
