# Supplementary figures and images for: The Administration of Circulating Extracellular Vesicles Modified by Anesthesia and Surgery Induces Delirium‐Like Behaviors in Aged Mice
Source: CNS Neurosci Ther. 2025 Jun 19;31(6):e70483. doi: 10.1111/cns.70483 (PMC12178831; doi:10.1111/cns.70483)

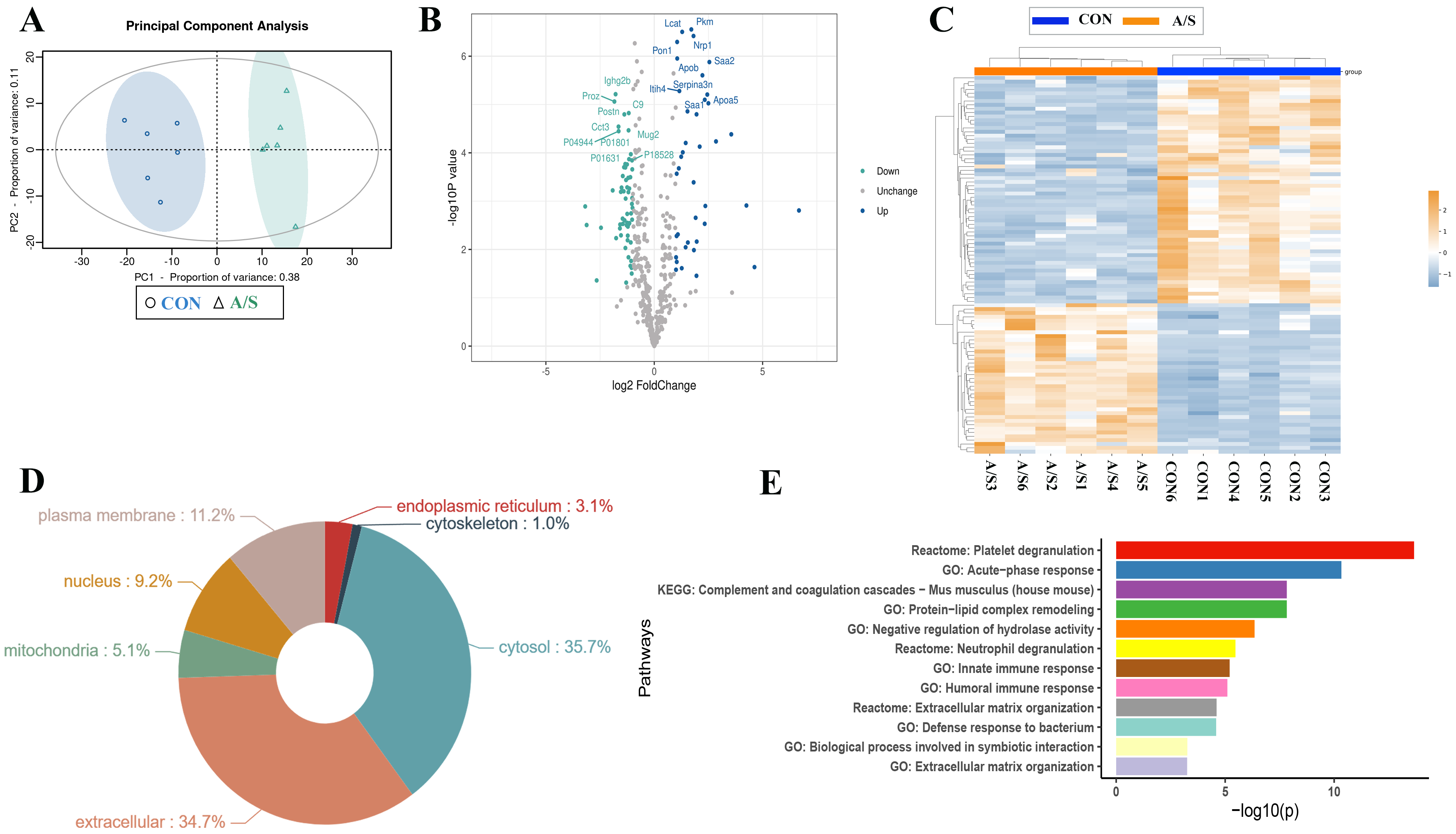

Supplement: Supplementary file 1 — Appendix S1. [file CNS-31-e70483-s001.zip › cns70483-sup-0003-FigureS1.tif]

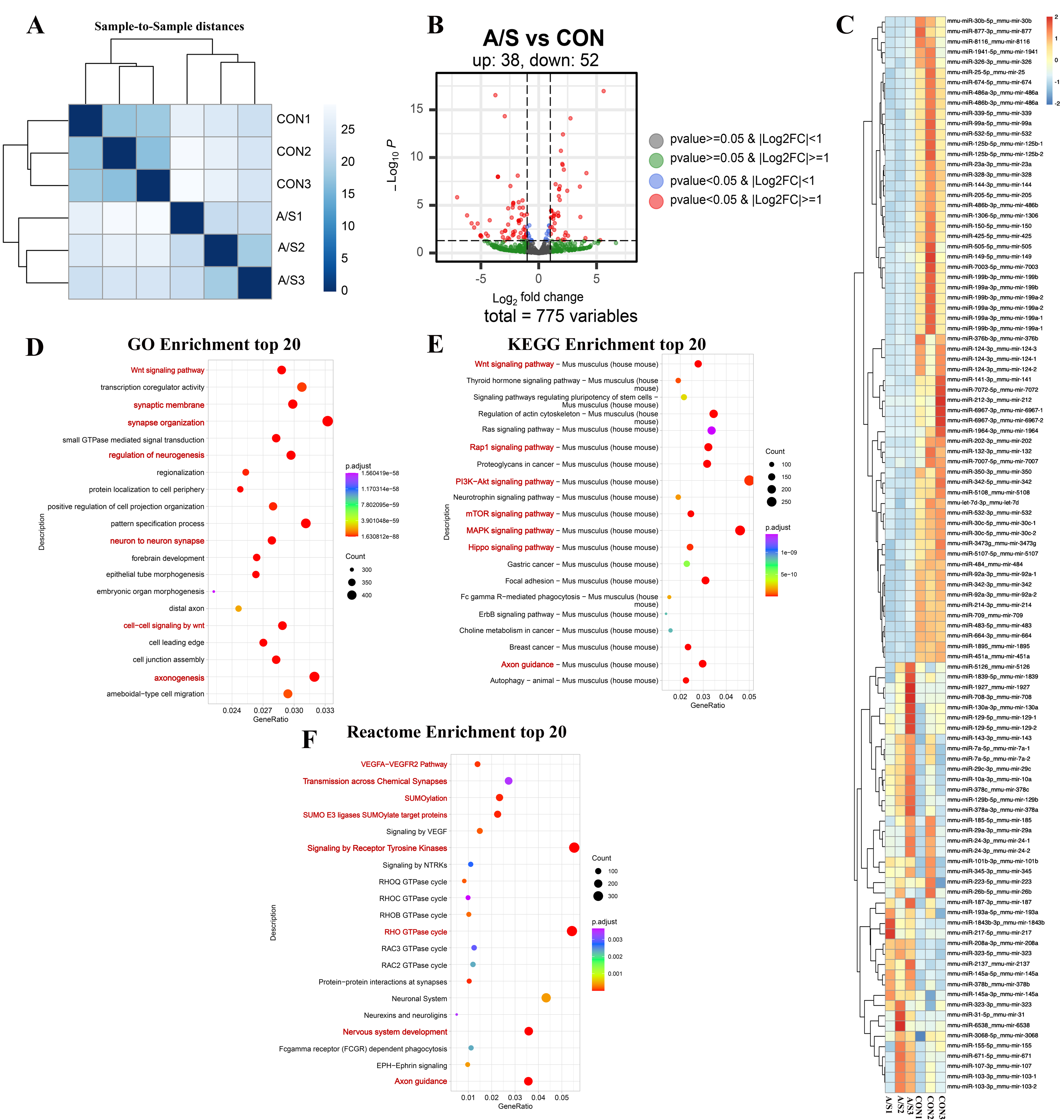

Supplement: Supplementary file 1 — Appendix S1. [file CNS-31-e70483-s001.zip › cns70483-sup-0004-FigureS2.tif]
